# Associations between the New DNA-Methylation-Based Telomere Length Estimator, the Mediterranean Diet and Genetics in a Spanish Population at High Cardiovascular Risk

**DOI:** 10.3390/antiox12112004

**Published:** 2023-11-15

**Authors:** Oscar Coltell, Eva M. Asensio, José V. Sorlí, Carolina Ortega-Azorín, Rebeca Fernández-Carrión, Eva C. Pascual, Rocío Barragán, José I. González, Ramon Estruch, Juan F. Alzate, Alejandro Pérez-Fidalgo, Olga Portolés, Jose M. Ordovas, Dolores Corella

**Affiliations:** 1 Department of Computer Languages and Systems, Universitat Jaume I, 12071 Castellón, Spain; 2 CIBER Fisiopatología de la Obesidad y Nutrición, Instituto de Salud Carlos III, 28029 Madrid, Spain; eva.m.asensio@uv.es (E.M.A.); carolina.ortega@uv.es (C.O.-A.); ignacio.glez-arraez@uv.es (J.I.G.); restruch@clinic.cat (R.E.);; 3 Department of Preventive Medicine and Public Health, School of Medicine, University of Valencia, 46010 Valencia, Spain; 4 Department of Internal Medicine, Institut d’Investigacions Biomèdiques August Pi Sunyer (IDIBAPS), Hospital Clinic, University of Barcelona, 08036 Barcelona, Spain; 5 Departamento de Microbiología y Parasitología, Facultad de Medicina, Universidad de Antioquia, Medellín 050010, Colombia; 6 Facultad de Medicina, Centro Nacional de Secuenciación Genómica—CNSG, Sede de Investigación Universitaria—SIU, Universidad de Antioquia, Medellín 050010, Colombia; 7 Department of Medical Oncology, University Clinic Hospital of Valencia, 46010 Valencia, Spain; japfidalgo@msn.com (A.P.-F.);; 8 Biomedical Research Networking Centre on Cancer (CIBERONC), Health Institute Carlos III, 28029 Madrid, Spain; 9 INCLIVA Biomedical Research Institute, 46010 Valencia, Spain; 10 Nutrition and Genomics, JM-USDA Human Nutrition Research Center on Aging at Tufts University, Boston, MA 02111, USA; 11 Nutritional Control of the Epigenome Group, Precision Nutrition and Obesity Program, IMDEA Food, UAM + CSIC, 28049 Madrid, Spain

**Keywords:** telomere length, DNA methylation, aging, Mediterranean diet, antioxidants, fruits, genetics, GWAS, epigenetics

## Abstract

Biological aging is a relevant risk factor for chronic diseases, and several indicators for measuring this factor have been proposed, with telomere length (TL) among the most studied. Oxidative stress may regulate telomere shortening, which is implicated in the increased risk. Using a novel estimator for TL, we examined whether adherence to the Mediterranean diet (MedDiet), a highly antioxidant-rich dietary pattern, is associated with longer TL. We determined TL using DNA methylation algorithms (DNAmTL) in 414 subjects at high cardiovascular risk from Spain. Adherence to the MedDiet was assessed by a validated score, and genetic variants in candidate genes and at the genome-wide level were analyzed. We observed several significant associations (*p* < 0.05) between DNAmTL and candidate genes (*TERT*, *TERF2*, *RTEL1*, and *DCAF4*), contributing to the validity of DNAmTL as a biomarker in this population. Higher adherence to the MedDiet was associated with lower odds of having a shorter TL in the whole sample (OR = 0.93; 95% CI: 0.85–0.99; *p* = 0.049 after fully multivariate adjustment). Nevertheless, this association was stronger in women than in men. Likewise, in women, we observed a direct association between adherence to the MedDiet score and DNAmTL as a continuous variable (beta = 0.015; SE: 0.005; *p* = 0.003), indicating that a one-point increase in adherence was related to an average increase of 0.015 ± 0.005 kb in TL. Upon examination of specific dietary items within the global score, we found that fruits, fish, “sofrito”, and whole grains exhibited the strongest associations in women. The novel score combining these items was significantly associated in the whole population. In the genome-wide association study (GWAS), we identified ten polymorphisms at the suggestive level of significance (*p* < 1 × 10^−5^) for DNAmTL (intergenics, in the *IQSEC1*, *NCAPG2*, and *ABI3BP* genes) and detected some gene–MedDiet modulations on DNAmTL. As this is the first study analyzing the DNAmTL estimator, genetics, and modulation by the MedDiet, more studies are needed to confirm these findings.

## 1. Introduction

Numerous studies have shown an important relationship between diet and chronic diseases [1,2,3,4]. The magnitude of the association varies depending on the disease type analyzed, which is generally higher for cardiovascular diseases, diabetes, metabolic syndrome, and obesity [1,2,3,4,5,6,7,8,9,10]. However, the precise mechanisms by which diet may exert a protective or risk effect against various chronic diseases are not well understood [11]. In general, oxidative stress is crucial in the pathogenesis, progression, and associated complications of the most prevalent chronic diseases [12,13,14,15]. An imbalance between the generation of reactive oxygen species and the antioxidant defense mechanisms typically causes cellular damage and dysfunction. Given that dietary foods can provide a variety of compounds with high antioxidant capacity, this is one of the postulated mechanisms for the protection of certain dietary patterns against these diseases [16,17,18].

In chronic diseases, another common factor in them is age, since the prevalence of these diseases increases with chronological age [19]. Nonetheless, it is widely recognized that a substantial distinction exists between chronological age, which refers to the time that has passed since birth, and biological age, which is a more complex concept developed from pathophysiological assessments that reflect the extent of an individual’s aging process [20,21]. Accelerated aging is the term used to describe the gap between chronological age and biological age [21,22].

The importance of oxidative stress in aging has been demonstrated in several studies [23,24], and there is a growing interest in identifying lifestyle factors (mainly diet) that may potentially mitigate the effects of biological aging in human studies [25,26]. The aging process is a multifaceted phenomenon primarily characterized by impairment in cellular, tissue, and organ functionality, leading to an augmented susceptibility to various diseases. This process encompasses a number of alterations, commonly referred to as “hallmarks of aging” [27]. These hallmarks are being characterized in depth [27,28]. In a recent update, the initial listing expanded from nine to twelve interconnected hallmarks [29]. Among them, we highlight “telomere attrition”, as it can be analyzed more easily in epidemiological studies in humans compared to other hallmarks such as “depletion of stem cells” or “modified intracellular communication” that require greater support of basic laboratory research.

Located at the ends of eukaryotic chromosomes, telomeres are specialized nucleoprotein structures with a protective function [30]. Telomeres consist of repetitive TTAGGG DNA sequences and are associated with a complex of six proteins referred to as Shelterin that includes the telomere repeat factor 1 (TRF1) and 2 (TRF2), protection of telomeres-1 (POT1), TIN2 (TRF1-Interacting Nuclear factor 2), Ras-related protein 1 (RAP1), and the telomere protection protein 1 (TPP1) [30,31]. The DNA component of telomeres undergoes a gradual reduction in length with every cycle of cell division, ultimately leading to the initiation of cellular senescence [30,31,32]. Multiple humans studies have observed that telomere length (TL) decreases with chronological age [33,34]. Similarly, a shorter TL has been associated with a higher risk of cardiovascular disease, mortality and other metabolic diseases [35,36,37,38,39,40]. Therefore, in epidemiological studies, TL has been proposed as a measure of biological aging despite the fact that measuring telomeres can be challenging [30,41]. Although TL may be different depending on the tissue analyzed, it has been shown that examining TL in peripheral leukocytes can be a good overall indicator [42]. The main drawback is the heterogeneity of the classical methodologies used to measure TL in epidemiological studies (i.e., terminal restriction fragment analysis, quantitative polymerase chain reaction, quantitative fluorescence in situ hybridization methods, or single telomere length analysis), which produce varying results [43,44,45]. Using the novel omics methods, Lu et al. developed and validated a new method to measure TL in epidemiological studies [46]. This method is based on the DNA methylation profile of specific cytosine phosphate guanine (CpG) sites derived by machine learning [46]. The DNA methylation estimator of TL (DNAmTL), based on 140 CpGs and validated in leukocytes from several cohorts, outperformed several limitations of the classical techniques in epidemiological studies [46]. Subsequently, this DNAmTL biomarker has been used in other cohorts [47,48,49,50,51], although more studies in diverse populations are needed to better know its external validity.

Moreover, as far as we know, none of the studies published with this biomarker of TL have analyzed the influence of the Mediterranean diet (MedDiet) on it. The MedDiet is recognized as a healthful eating pattern that is high in antioxidants and can have anti-aging benefits [11,52,53,54]. The traditional MedDiet is a dietary pattern that was commonly observed in Greece and Italy throughout the early 1960s [55]. Furthermore, it is important to remember that in Spain, individuals also followed the typical MedDiet [11]. From the initial definition of the MedDiet to the spread of this diet internationally, mainly in the United States, thanks to the work of Dr. Dimitrios Trichopoulos [56] and Dr. Antonia Trichopoulou [57], there has been extensive research and dissemination activity to the general population, characterizing this diet as one of the healthiest in the world. A further detail of this historical evolution of the MedDiet can be found in the review by Sikalidis et al. [55]. The traditional MedDiet is characterized by a preferential use of virgin olive oil, seasonal vegetables, fruits, legumes, nuts and seeds, low red meat consumption and moderate fish consumption [11]. It has been indicated that the overall dietary pattern rather than individual components is important for health and longevity [56]. Thus, the MedDiet can reduce the level of oxidative stress markers and inflammation due to a high abundance of antioxidant compounds such as ω-3 fatty acids and resveratrol, among others [11]. It is important to note that the first meta-analyses on the topic already associated the MedDiet with greater longevity [57] as well as with a lower mortality from all causes and specific causes [58].

However, previous studies examining the association between the MedDiet and telomere length have yielded varying results [59,60,61,62,63,64,65,66,67,68,69,70,71,72,73,74,75]. Despite the conclusion of a meta-analysis that higher adherence to the MedDiet was related to longer TL, the association was heterogeneous, and sex differences were observed [72] with women having more significant associations than men. On the other hand, it is known that genetic factors play a relevant role in TL [76,77,78]; however, the vast majority of previous studies analyzing the impact of the MedDiet on TL did not account for genetic variants [59,60,61,62,63,64,65,66,67,68,69,70,71,72,73,74,75]. Moreover, although multiple GWASs have been taken for TL measured with the classic techniques [76,77,78,79,80,81,82,83,84,85,86], there are barely any GWAS using the DNAmTL variable [51,87]. Thus, little is known about the genetic architecture underlying DNAmTL, and more research is needed. In this study, we first aimed to investigate the association between adherence to the MedDiet and TL in a Spanish Mediterranean population using the DNAmTL estimator, taking into account sex-specific analyses. Our secondary aims were to perform an exploratory GWAS on DNAmTL to identify genetic variants associated with this biomarker and to explore gene–MedDiet modulations on DNAmTL both at the TL-related candidate genes and at the genome-wide level.

## 2. Materials and Methods

### 2.1. Participants and Study Design

A total of 414 individuals, comprising both men and women, aged between 55 and 75 years, were analyzed in the present study. These individuals were recruited in the PREDIMED-Plus-Valencia study. The PREDIMED-Plus-Valencia study is one of the field sites for the ongoing multicenter PREDIMED-Plus trial [88]. The participants in this study were selected from primary health care facilities in the Valencia region according to the inclusion and exclusion criteria outlined in the PREDIMED-Plus trial protocol. The Valencia region is situated along the eastern Mediterranean coast of Spain. The subjects selected for participation in the study were adults living in the community, with a body mass index (BMI) ranging from 27 to 40 kg/m^2^ and exhibiting at least three components of the metabolic syndrome [88]. A total of 465 participants were recruited at the PREDIMED-Plus Valencia site. However, for the purposes of this study, 414 subjects were included. This number represents individuals who consented to participate in the genomics/epigenomics studies and whose DNA samples and methylation workflows met the quality control criteria for the leukocyte DNA methylation analysis, as previously reported [89]. A cross-sectional study using baseline (sociodemographic, dietary, lifestyle, clinical, epigenetic and genetic) data was undertaken. The Human Research Ethics Committee of the University of Valencia provided ethical approval in accordance with the Helsinki Declaration for the study protocols and procedures (approval codes H1373255532771, 15 July 2013; and H1509263926814, 6 November 2017). Written informed consent was obtained from the participants.

### 2.2. Clinical, Biochemical and Anthropometric Variables

At baseline, demographic information, clinical variables, and medication use were assessed by questionnaire as previously reported [90]. Following a 12 h overnight fast, plasma glucose, total cholesterol, HDL-C, LDL-C, and triglyceride concentrations were measured as previously reported [91]. In addition, we assessed the complete blood count (CBC) in peripheral blood samples. The CBC included the total number of leukocytes as well as the type of white blood cells (neutrophils, eosinophils, basophils, monocytes, and lymphocytes) as previously indicated [92]. Blood pressure was measured by qualified personnel using the study protocol and a validated semiautomatic oscillometer (Omron HEM-705CP, Hoofddorp, The Netherlands) [88]. Anthropometric data (height, weight, and waist circumference) were measured by qualified personnel using calibrated scales, a standardized ribbon, and a wall-mounted stadiometer as previously reported [88]. The BMI was calculated by dividing the individual’s weight in kilograms by their height in meters squared. The condition of obesity was operationally defined as having a BMI equal to or exceeding 30 kg/m^2^.

### 2.3. Adherence to the MedDiet and Other Lifestyle Factors

The validated PREDIMED-Plus 17-item score [93], an updated version of the previously described PREDIMED 14-item scale [94], was used to evaluate adherence to the MedDiet. This 17-item questionnaire contained seventeen questions about the MedDiet. The questionnaire was scored as follows: 1 point for each response that captured adherence to the MedDiet, and 0 points for responses that did not. Table S1 displays the full set of questions and responses for MedDiet adherence. In brief, the foods and habits included were as follows: I-1: use only extra virgin olive oil for cooking, salad dressings, and spreads; I-2: vegetable consumption; I-3: fruits; I-4: red meat; I-5: butter, margarine or cream; I-6; sugar-sweetened beverages; I-7: legumes; I-8: fish/shellfish; I-9: pastries, cookies, sweets or cakes; I-10; nuts; I-11: preference for white meat; I-12: “sofrito” (Mediterranean sauce made with tomato, onion, garlic and olive oil); I-13: add preferentially non-caloric artificial sweeteners to beverages instead of sugar; I-14: servings of white bread; I-15: whole grains; I-16: refined cereals; I-17; moderate wine consumption. The greater the score (0 to 17), the greater the adherence to the MedDiet pattern. As previously reported [92], the overall score was categorized into two groups representing low (from 0 to 8 points) and high (from 9 to 17 points) adherence to the MedDiet. Both the overall score and specific food items were analyzed. In addition, a previously described PREDIMED-Plus general questionnaire [88] administered by trained personnel at baseline collected information regarding tobacco use and educational attainment. Those classified as current smokers smoked at least one cigarette, cigar, or a pipe per day. There defined three categories for smoking status: current smokers, ex-smokers, and non-smokers. Lastly, never-smokers and ex-smokers were compared to current smokers. Using the validated REGICOR Short Physical Activity Questionnaire [95], the estimation of total energy expenditure related to leisure-time physical activity involved the summation of the frequency, duration, and intensity of each activity, which was then divided by the number of days in a month (30 days) to provide the value in METmin/day.

### 2.4. DNA Isolation, DNA Methylation and DNAmTL Calculation

At baseline, DNA was extracted from as described in a prior publication [92]. Double-stranded DNA was quantified using PicoGreen (Invitrogen Corporation, Carlsbad, CA, USA). A subsequent investigation of leukocyte DNA methylation was conducted only on samples containing 500 ng of DNA of good quality. In this study, we employed the Infinium HumanMethylationEPIC BeadChip (850K) array, which was manufactured by Illumina in San Diego, CA, USA. This array allows for the analysis of methylation patterns by examining approximately 850,000 CpG sites. Notably, it covers more than 90% of the probes detected on the 450 K array and includes extra CpG sites [96]. In order to minimize batch effects, the position (sample wells) of the DNA samples on the microarrays were randomized [97]. The arrays underwent additional processing at the Human Genomics Facility located at Erasmus MC in Rotterdam, the Netherlands. The DNA was processed by bisulfite conversion using the Zymo EZ-96 DNA Methylation Kit (Zymo Research, Irvine, CA, USA) and subsequently hybridized to the Illumina EPIC array, following the instructions provided by the manufacturer. The microarrays underwent scanning using an Illumina HiScan machine, resulting in the generation of “*.idat” files. The Human Genomics laboratory conducted quality control processes to evaluate the quality and reliability of the DNA methylation data obtained. These procedures involved utilizing the R packages Minfi (release 3.18), Meffil (release 1.1.1), and ewastools (release 1.7.2) [98,99,100]. In summary, the quality control process aimed to identify samples that had deficiencies or substandard control metrics, specifically in relation to inadequate bisulfite conversion, various forms of suboptimal hybridization, and samples with a low call [92]. A total of 414 samples successfully underwent quality control and were thereafter subjected to analysis for the computation of DNAmTL. Data normalization, including functional normalization and normal-exponential out-of-band (NOOB) correction, was carried out. [92,101]. Beta values were obtained for the corresponding CpG sites [92]. The resulting DNAm beta matrix was uploaded to the online Horvath epigenetic age calculator [102]. The Horvath DNAmTL was used for TL estimation as previously published by Lu et al. [46]. For this estimator, methylation at 140 specific CpG sites are used to derive TL measures in kilobases (kb) according to the initial machine learning model of telomere restricted fragments (TRF)-measured leukocyte TL [46]. Likewise, the age-adjusted estimate of DNAmTL (referred to as DNAmTLadjAge) was derived online by regressing DNAmTL on age, and the resulting raw residual was defined as DNAmTLadjAge [46]. In addition, the same DNAm data were used to calculate the intrinsic epigenetic age acceleration (IEAA) for the Hannum epigenetic clock [103] to estimate the correlation between both epigenetic measures. IEAA is defined as the residual resulting from regressing the DNAm age estimate from Hannum on chronological age and blood cell count estimates [104]. Positive IEAA indicates a higher biological age than the chronological age, whereas negative IEAA values indicate a lower biological age than expected [105]. Furthermore, we assessed the quality of the methylation computations by determining whether the methylation beta value distributions deviated from a gold standard (corSampleVSgoldstandard > 0.80) [102,104]. All 414 samples passed the criterion at >0.90.

### 2.5. Genetic Analysis

From the isolated DNA of the 414 participants, the University of Valencia conducted high-density genotyping utilizing the Infinium OmniExpress-24 v1.2 BeadChip genotyping array, which captured 713,599 markers (Illumina Inc., San Diego, CA, USA). The genotyping was performed following the manufacturer’s methodology and adhering to previously stated quality criteria [106]. The Genome Studio genotyping module (Illumina, Inc.) was utilized for allele identification and genotype determination. Using conventional analysis pipelines implemented in the Python programming language with the Numpy library modules and PLINK [107,108], cleaning of data was performed. Single nucleotide polymorphisms (SNPs) non-mapped on autosomal chromosomes, SNPs with a minor allele frequency (MAF) of less than 0.01, SNPs that differed from the anticipated Hardy–Weinberg equilibrium with a *p*-value less than 1.0 × 10^−4^, and SNPs with a call rate lower than 90% were excluded [106]. After genotyping, we employed two approaches for genetic analysis. In the first, we selected directly measured SNPs in candidate genes previously linked to TL across several GWAS [109]. In the second approach, we explored the association between the genome-wide measured SNPs and DNAmTL in this population. In addition, we conducted post-GWAS analyses utilizing FUMA (Functional Mapping and Annotation) [110] and FORGE2 [111,112] tools.

### 2.6. Statistical Analyses

First, descriptive statistical analyses were conducted. Proportions were compared using Chi-square testing. The mean values of continuous variables were compared using Student’s t-tests and ANOVA testing. The concentrations of triglycerides were subjected to a logarithmic transformation in order to carry out statistical testing. The association analysis involved several regression models depending on the variable characteristics. We analyzed the raw association between DNAmTL (as continuous) and chronological age at time of blood collection in the whole population and by sex using linear regression. Scatter plots were displayed. Several multivariate regression models were gradually fitted to account for potential confounders, including sociodemographic, clinical, anthropometric and lifestyle variables and DNAmTL as a continuous variable. When considering two categories of TL based on the DNAmTLadjAge, we also employed logistic regression. We placed negative DNAmTLadjAge values in the category of shorter than expected TL based on age, and we placed positive values in the opposite category (longer than expected). As specified in the corresponding analyses, multivariate logistic models were fitted sequentially. The association between SNPs in candidate genes previously associated with TL in multiple GWAS [83] and DNAmTL was analyzed using sex- and age-adjusted linear regression. The genotypes were analyzed using an additive genetic model that considered the minor allele for each SNP. Likewise, the association between DNAmTL and the methylation beta values of each CpG site included in the estimator of Lu et al. [46] was analyzed by multivariate lineal regression models.

Adherence to the MedDiet was examined as continuous as well as categorical when indicated. We also tested the associations with the specific foods of the MedDiet score and derived novel sub-scores based on the results. Statistical analyses were conducted for the entire population and stratified per sex. Additionally, the sex heterogeneity was tested by computing the statistical significance of the interaction term between sex and the variable of adherence to the MedDiet in the corresponding model described in the results.

For the exploratory GWAS to identify genes and SNPs associated with DNAmTL in this population, we fitted general linear models in PLINK [107,108]. Each SNP at the genome-wide level was considered in accordance with an additive pattern, and multivariable adjustment for sex, age and other variables specifically indicated in each model was undertaken. Regression coefficients for the minor allele for each SNP were estimated. For the SNP-based GWAS, we used the conventional threshold of *p* < 5 × 10^−8^ for genome-wide statistical significance. Likewise, SNPs with *p*-values below 1 × 10^−5^ were also considered suggestive of genome-wide significance. Despite these general considerations, as there are very few GWAS studies that analyzed DNAmTL as the outcome, some tables show SNPs with *p*-values above this threshold for meta-analysis. A quantile–quantile plot (Q-Q plot) comparing the expected and observed *p*-values was performed in the R-statistical environment [113], and we used R qqman R library to create Manhattan plots. Despite the fact that all participants were white Caucasians, and no ethnic bias or population stratification was anticipated, we computed the genomic inflation factor (lambda coefficient). Likewise, we adjusted for the top of the principal components derived in the population stratification analysis as previously reported [106]. We used LocusZoom.js to generate locus-specific graphical displays of the position of the selected SNPs in the GWAS to nearby genes and local recombination hotspots [114] as well as to indicate the linkage disequilibrium (LD). In addition to the SNP-based GWAS, we carried out a gene-based GWAS with FUMA [110]. This analysis considers the aggregate effect of multiple SNPs in a single test instead of analyzing each SNP separately [110]. The FUMA platform uses MAGMA (Multi-marker Analysis of GenoMic Annotation) [115] as well as the annotation of the selected genes in biological context [110,116]. In addition, we used FORGEdb, a resource of genomic annotations and an integrated score of functional tools [111,112], to rank the selected loci into biological insight. Finally, we explored some gene–MedDiet interactions in determining DNAmTL. Adherence to the MedDiet was considered as dichotomous as previously reported [92,117]. We chose “a priori” the lead candidate gene SNP for TL, associated with DNAmTL in this population, and examined the significance of the SNP–MedDiet interaction term in a hierarchical multivariable regression model. We also conducted an exploratory genome-wide SNP–MedDiet interaction [117] on DNAmTL. For analyses not involving genome-wide associations, a two-sided *p*-value 0.05 was considered statistically significant.

## 3. Results

### 3.1. Participants Characteristics

Participants were 414 individuals (184 men and 228 women) recruited for the PREDIMED-Plus Valencia study and having high-quality DNA methylation data at the baseline visit. The demographic, clinical, anthropometric, and lifestyle characteristics are presented in Table 1.

They were elderly (mean age 65.7 ± 0.2 years at the baseline visit) individuals with metabolic syndrome. We presented the mean age at the recruitment visit in addition to the age at the baseline visit after a run-in period included in the study protocol [88]. During the baseline visit, DNA methylation analysis and all other measurements were performed. The mean BMI was high (32.3 ± 0.2 kg/m^2^) and did not differ significantly between sexes. There were no sex differences in fasting glucose, medication use, or adherence to the MedDiet. Nonetheless, some variables, including age, total HDL-C, physical activity, educational level, and tobacco use, varied by sex. In the whole population, the prevalence of current, former, and never smokers was 11.6%, 43.0%, and 45.4%, respectively. In addition to the quantitative variable (17 points score) for adherence to the MedDiet, we considered a dichotomous variable indicating high (≥9 points) or low adherence (<9 points) to the MedDiet. No significant differences (*p* = 0.648) in prevalence were detected in men (40.3% high adherence) versus women (42.5% high adherence).

### 3.2. Descriptive Measures of DNAmTL in this Population

The DNAmTL (raw data) for the whole population was 6.6958 ± 0.0172 kilobases (kb) (Table 2). This estimator possesses the same units (kb) as that of the mean measured TL by the gold standard method of TRF [46]. DNAmTL (raw data) was statistically higher in women than in men (*p* = 0.025). Moreover, when DNAmTL was adjusted for age (referred to as DNAmTLAdj/Age), this difference by sex was more prominent (*p* < 0.001) (Table 2). From the calculated DNAmTLAdj/Age, we obtained the number of subjects having shorter TL. A negative value of DNAmTLAdj/Age would indicate DNAmTL that is shorter than expected (53.8% in men versus 41.2% in women; *p* = 0.013), while a positive value would indicate higher DNAmTL than expected based on age. We also presented in Table 2 another descriptive measure of epigenetic age acceleration (IEAA Hannum) in this population. The mean value of this biomarker was positive in men (indicating higher biological age than the chronological age), whereas a negative mean value for IEAA was found in women, indicating lower biological age than expected (*p* = 0.011).

**Figure 1:**
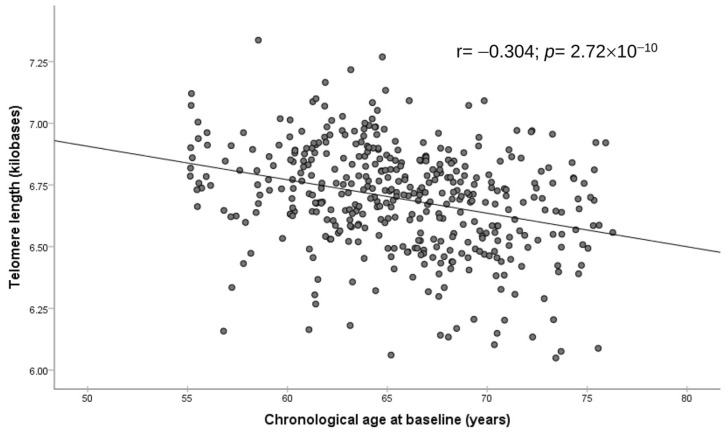
Association between leukocyte telomere length–DNA methylation and chronological age in the whole population (*n* = 414). Scatter plots with raw values, Pearson correlation coefficient and *p*-value.

Figure 1 displays the correlation between chronological age at baseline in the whole population (age at the time of blood collection) and DNAmTL (raw values). We observed an inverse association (r = −0.304; *p* = 2.72 × 10^−10^). The corresponding regression coefficient indicated that DNAmTL shortened by 0.014 kb per year (95%CI: −0.018 to −0.009; *p* = 2.72 × 10^−10^).

**Figure 2:**
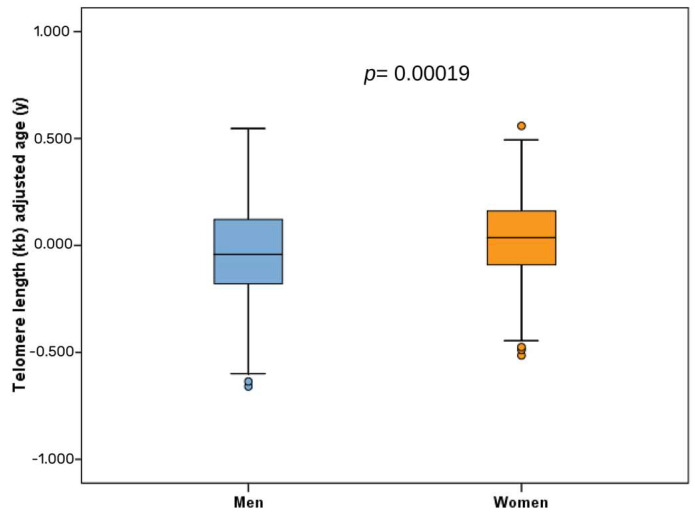
Boxplot of telomere length–DNA methylation adjusted for age (DNAmTLAdjAge) in men and women (*n* = 414). Raw data of the age-adjusted variable (DNAmTLAdjAge) per sex and the corresponding *p*-value for the comparison of means between men and women.

Similar inverse correlations were observed when men and women were analyzed separately (Figure S1, panels A and B). Figure 2 shows the boxplots of DNAmTLAdj/Age in men and women.

Moreover, we detected an expected inverse association between DNAmTL and the other biomarker of age acceleration, the IEAA-Hannum (r = −0.465; *p* = 1.5 × 10^−23^ for DNAmTL and r = −0.307; *p* = 1.9 × 10^−10^ for DNAmTLAdj/Age), adding validity to this TL estimator.

Next, we analyzed the site-specific association between the 140 CpGs contributing to the estimator [46] and the derived DNAmTL in this population. Table 3 displays the 10 most statistically significant CpG sites associated with the global DNAmTL.

As specified, models were multivariable adjusted, and the partial correlation coefficient for each CpG site indicated an inverse or direct correlation between methylation at that site and DNAmTL. The most significant CpG associated with DNAmTL was located on chromosome 4 within the *NCAPG* (Non-SMC Condensin I Complex Subunit G) gene. Hypermethylation of this site was associated with longer DNAmTL. Likewise, hypermetilation at cg14391737 in the *CCND3* (Cyclin D3) gene and at the cg01940273 in the *TPI1* (Triosephosphate Isomerase 1) gene were also associated with longer DNAmTL. Table S2 provides additional information on the top-ranked CpGs, including other CpG sites associated with DNAmTL.

### 3.3. Associations between SNPs in Candidate Genes Related to TL and DNAmTL

Here, we tested the association between SNPs in candidate genes previously associated with telomere length [83] and the estimated DNAmTL in this population. Table 4 shows these associations sorted by statistical significance and including the 20 most significant SNPs.

The corresponding regression coefficients (beta) refer to the least common allele, and models were adjusted for sex and age. The most significant SNP was rs2075786 in the *TERT* gene (telomerase reverse transcriptase) with *p* = 0.008. The TL in carriers of the minor allele was 0.0407 kb shorter (per allele) compared to the homozygote for the frequent allele. The second most significant SNP was rs3785073 located in the *TERF2* gene (telomeric repeat binding factor 2) with *p* = 0.0086. For this SNP, the minor allele was also associated with a shorter TL. Statistically significant results have also been obtained for SNPs in the *RTEL1* (regulator of telomere elongation helicase 1) and *DCAF4* (DDB1 and CUL4 associated factor 4) genes. These significant associations with genes associated with TL contribute to increase the validity of the DNAmTL estimator derived in this population.

In addition, we computed a genetic risk score (GRS) by aggregating the effects of the four candidate genes that were found to be statistically significant. The most significant SNP variants of each gene were chosen for the GRS (*TERT*-rs2075786, *TERF2* rs3785073, *RTEL1*-rs6011011, and *DCAF4*-rs17781739) with only one SNP per gene examined. The effect allele linked to a shortened DNAmTL was accounted for when calculating the score. The GRS ranged from 1 to 7. However, the prevalence of the scores 1 and 7 was low. Then, scores 1 and 2 were computed in the same category. Likewise, scores 6 and 7 were considered together. This GRS was strongly associated with the DNAmTL (*p* < 0.001). Figure S2 shows the association of this score and DNAmTLAdj/Age in a model additionally adjusted for diabetes and BMI.

### 3.4. Association between Adherence to the MedDiet and Shorter DNAmTL

Following the genetic validation of the DNAmTL estimator, we examined the association between adherence to the MedDiet (as a continuous variable) and DNAmTL, taking into account two distinct categories: subjects whose TL was shorter than expected and subjects whose TL was longer than expected (Table 5). Odds (OR and 95% CI) of having a shortened TL associated with a one-point increase in the MedDiet 17-I score were calculated utilizing multivariable logistic regression. Three multivariable-adjusted models were fitted for the whole population and by sex as indicated. Greater adherence to the MedDiet was associated with a decreased likelihood of having a shorter TL for the entire population (OR = 0.92; 95%CI: 0.86–0.99; *p* = 0.032 in model 1). In the fully adjusted model (model 3), this association remained statistically significant (*p* = 0.049).

**Figure 3:**
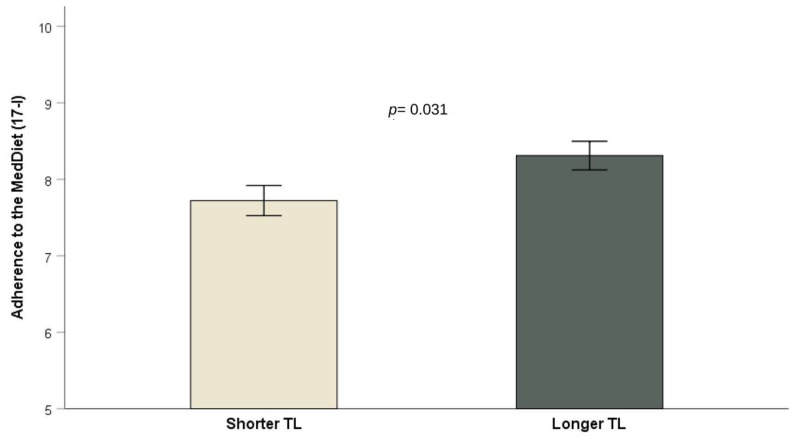
Adherence to Mediterranean diet (means ± SE) in subjects with shorter (*n* = 194) and longer (*n* = 220) TL. *p* = 0.031 for difference of means in a model adjusted for sex and age. *p* = 0.034 for difference of means in a model additionally adjusted for diabetes and BMI. *p* = 0.044 in the model additionally adjusted for metformin, insulin, lipid-lowering drugs, hypertension medication, systolic blood pressure, education, smoking, and physical activity. Error bars: SE of means.

The association, however, only attained statistical significance among women (OR = 0.88; 95%CI: 0.79–0.98; *p* = 0.023 for model 3) in the stratified analysis by sex. As shown in Figure 3, individuals with shorter TL exhibited lower levels of adherence to the MedDiet score when compared to those with longer TL (*p* < 0.05).

### 3.5. Association between Global Adherence to the MedDiet and Consumption of Particular Items with DNAmTL (as a Continuous Variable)

Furthermore, we examined the association between adherence to the MedDiet and DNAmTL as a continuous variable. Table 6 shows the results in the whole population and stratified per sex.

**Figure 4:**
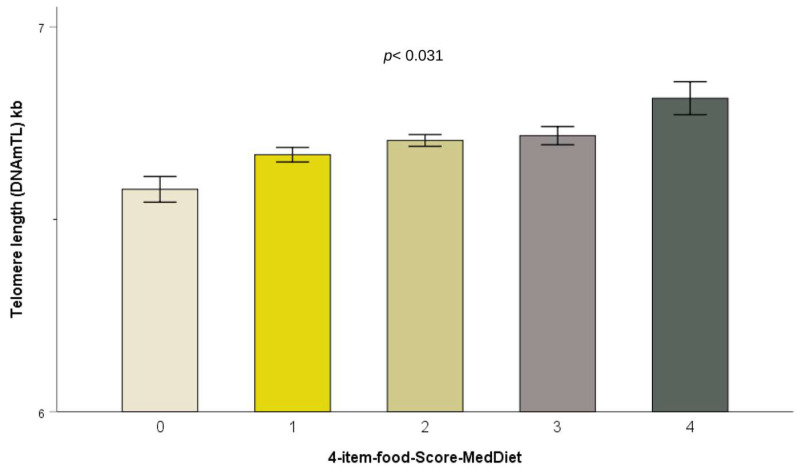
Telomere length (DNAmTL) depending on the adherence to the 4-MedDietFood score ranging from 0 to 4. Values are adjusted mean ± SE. *p* < 0.001 for the lineal trend of the 4-MedDietFood score in Models 1, 2 and 3. Error bars: SE of means.

In women, the global score of adherence to the MedDiet (17-I) was found to be significantly associated with longer DNAmTL (*p* = 0.002 in Model 1 after adjusting for age, and this association persisted in multivariate Model 3 after controlling for age, diabetes, BMI, metformin, insulin, lipid-lowering drugs, hypertension medication, SBP, education, smoking, and physical activity). We found, according to Model 3 in women, that a one-point increase in the MedDiet adherence score resulted in a 0.015 ± 0.0005 kilobases increase in DNAmTL length. In the case of males, no statistically significant association was found. MedDiet was also evaluated as a dichotomous variable. In women, there was a significant association between higher adherence to the MedDiet (score ≥ 9) and longer DNAmTL (0.078 ± 0.026; *p* = 0.004) compared to lesser adherence. Although the MedDiet pattern is more important as a whole, we examined the particular foods/items of the MedDiet score. Table S3 shows the compliance for each item by sex according to the criteria for adherence to the MedDiet specified in Table S1. Table S4 shows the statistical significance of the associations between each one of the 17 MedDiet items and DNAmTL by sex. In women, we detected statistically significant associations (*p* < 0.05) with daily fruit intake, fish intake, “sofrito” and whole grain intake. Compliance with the criteria predefined for adherence to the MedDiet for each item was associated with significantly longer DNAmTL. These associations remained statistically significant even after multivariate adjustment in Model 3. We then calculated a new MedDietFood score by adding these four items. The 4-MedDietFood score ranged from 0 to 4 points. Table 6 shows the association between this score and DNAmTL in the whole population and by sex. Beta coefficients for category 1 (zero points) in comparison with category 5 (4 points) were computed. We observed a reduction in DNAmTL in subjects who did not adhere to the four food items as compared to those who adhered completely; results were statistically significant for women and for the whole population in Model 3. In men, Models 1 and 2 were also statistically significant. Therefore, we considered this score for the whole population in further analyses. Figure 4 shows DNAmTL according to the score obtained in the 4-MedDietFood item variable in the whole population.

### 3.6. Exploratory GWASs for DNAmTL

**Figure 5:**
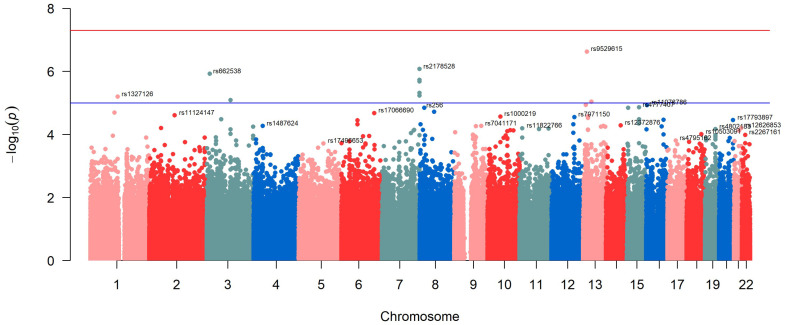
Manhattan plot of the GWAS of leukocyte telomere length–DNA methylation using a gene-based approach (adjusted for sex and age). The red line represents the threshold (−log_10_(5 × 10^−8^)). The blue line represents the threshold (−log_10_(1 × 10^−5^)).

To examine the SNPs associated with the DNAmTL in this population, we initially performed an exploratory SNP-based GWAS. The Manhattan plot corresponding to the model that was adjusted for age and gender is depicted in Figure 5. Figure S3 displays the Q-Q plot for this GWAS as well as the lambda parameter (lambda = 1.004), indicating no statistical inflation or population stratification bias. Ten SNPs surpassed the suggestive level of significance for GWAS (*p* < 1 × 10^−5^). Nevertheless, we were unable to identify any SNPs that were significantly associated at the genome-wide level (*p* < 5 × 10^−8^).

Table 7 shows the details corresponding to the most significantly associated SNPs with DNAmTL in Figure 5, ranked by *p*-values.

**Figure 6:**
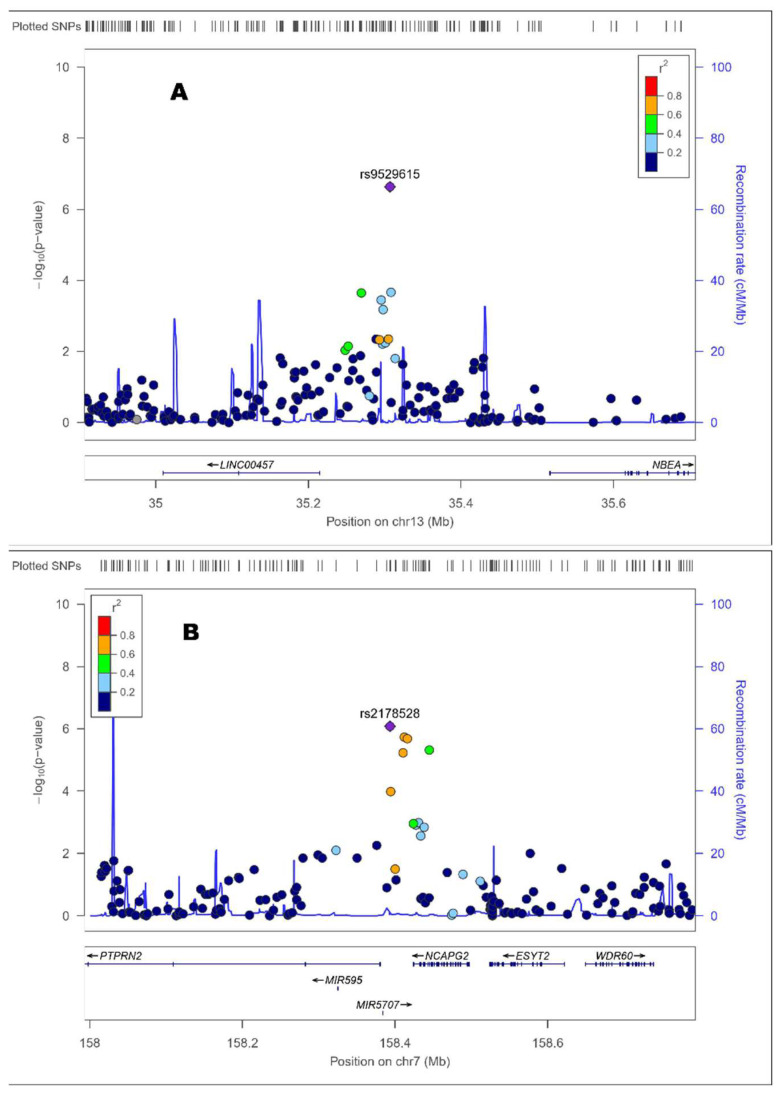
Zoom plots of the selected top-ranked SNPs for the GWAS of DNAmTL in this population (Table 5). The panel shows the following SNPs: (**A**) rs9529615 (Chr.: 13; *p* = 2.33 × 10^−7^); (**B**) rs2178528 (Chr.: 7; *p* = 8.30 × 10^−7^).

The hit was the rs9529615 SNP located in chromosome 13 at an intergenic position. The minor allele of this SNP was associated with higher DNAmTL (beta: 0.0726; *p* = 2.3 × 10^−7^). Figure 6 (panel A) shows the Zoom plot corresponding to the rs9529615 SNP intergenic in chromosome 13. The second most significant SNP was the rs2178528 in chromosome 7. The minor allele for this SNP was associated with shorted DNAmTl (beta: −0.0737; *p* = 8.3 × 10^−7^).

The Zoom plot (Figure 6, panel B) shows that this intergenic SNP is located near the *PTPRN2* (protein tyrosine phosphatase receptor type N2) gene and near the *NCAPG2* (Condensin-2 complex subunit G2) gene. Among the other 10 top-ranked SNPs surpassing the suggestive level for GWAS significance, we detected SNPs in the *IQSEC1* (IQ Motif and Sec7 Domain ArfGEF 1), the *NCAPG2* and the *ABI3BP* (ABI Family Member 3 Binding Protein) genes. Figure S4 presents the Zoom plots for the selected intergenic top-ranked SNPs: Panel A (rs11983468 in chromosome 7); Panel B (rs1327126 in chromosome 1); and Panel C (rs12427518 in chromosome 13). Table 7 also shows the FORGEdb Score for each SNP to rank the most significantly associated loci into biological insight. This score ranging from 0 to 10 provides information on associated regulatory elements, transcription factor (TF) binding sites and target genes. The FORGEdb scores are computed based on the presence or absence of five different lines of evidence for regulatory function: (I) DNase I hotspot, marking accessible chromatin (2 points); (II) Histone mark ChIP-seq broadPeak (2 points); (III) TF motif (1 point) and Contextual Analysis of TF Occupancy (CATO) score, marking potential TF binding (1 point); (IV) Activity-by-contact (ABC) interaction, marking gene looping (2 points); and (V) Expression quantitative trait locus (eQTL), marking an association with gene expression (2 points).

Table S5 provides a detailed summary of the global FORGEdb scores computed for the 10 most statistically significant SNPs. In general, these SNPs reached good FORGEdb scores. Among them, we would like to outline the rs12427518 that reached the maximum score of 10, suggesting a large amount of evidence for the functional relevance of this intergenic SNP located between the *OLFM4* (Olfactomedin 4) and the *PCDH8* (Protocadherin 8) genes.

In addition, to better understand the functionality of these SNPs and related genes, we carried out a functional analysis with the FUMA tools as indicated in the Methods section. Figure S5 shows the gene expression heatmap for the top-ranked selected genes (*IQSEC1*, *NCAPG2* and *PTPRN2*) using the GTEx V8 (54 tissue-types) dataset and showing the average expression per label (log2 transformed).

Additional adjustment of the model for additional potential confounders did not change significantly the list of the top-ranked SNPs. Table S6 presents the corresponding estimates in the GWAS for DNAmTL adjusted for sex, age, diabetes and BMI.

Next, we carried out a gene-based exploratory GWAS for DNAmTL in this population. Figure S6 displays the gene-based Manhattan plot for the corresponding GWAS. The model was adjusted for sex and age. The 14 most statistically significant genes were annotated. For a gene-based GWAS, the threshold 1 for statistical significance is set at (−log_10_(2.7 × 10^−6^)), considering the strict Bonferroni correction. Likewise, a threshold 2 (−log_10_(1 × 10^−4^)) at the suggestive level of statistical significance is considered. Several genes in our analysis neared the suggestive level of significance, but none achieved it.

However, we identified some genes, previously detected at the SNP level analysis, such as the *ABI3BP* or the *THSD4* (thrombospondin type 1 domain containing 4). But most importantly, we detected as top-ranked several genes previously characterized as genes being associated with telomeres; these genes include *ETHE1* (ETHE1 Persulfide Dioxygenase), *BRCA2* (Breast Cancer Type 2 Susceptibility) and *RFC2* (Replication Factor C Subunit 2) genes.

### 3.7. Exploratory Analysis of Gene–MedDiet Interactions in Determining DNAmTL

Finally, we explored the joint contribution of adherence to the MedDiet and the main genetic variants on DNAmTL to identify additive or interactive effects. First, we focused on candidate genes previously reported to be associated with TL (Table 4). In this exploratory analysis, we selected the SNP in candidate genes most statistically significantly associated with DNAmTL (rs2075786 in the *TERT* gene) and analyzed the interaction with adherence to the MedDiet (as dichotomous variable: low/high), analyzing the interaction term in a hierarchical model additionally adjusted for sex, age, BMI and diabetes. The interaction term did not reach the statistical significance (*p* = 0.428).

Focusing on the main effects, both the *TERT* SNP (*p* = 0.034) and the adherence to the MedDiet (*p* = 0.047) contributed independently and additively to the DNAmTL. The minor allele was associated with a decreased TL in comparison with the major allele. Thus, homozygous subjects for the minor allele had −0.087 ± 0.035 kb less in the mean DNAmTL than homozygous subjects for the major allele (*p* = 0.012). This difference can be reduced by a having a high adherence to the MedDiet (≥9 points). This variable was associated with an increase of 0.041 ± 0.020 kb in DNAmTL.

**Figure 7:**
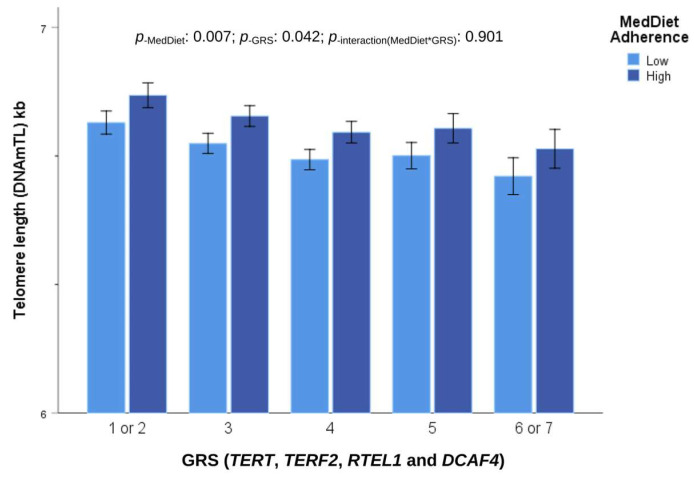
Combined effect of adherence to Mediterranean diet (MedDiet), expressed as categories (low and high), and genetics using a genetic risk score(GRS) of *TERT*, *TERF2*, *RTEL1* and *DCAF4*, with the effect allele associated with shorter telomere length on DNAmTL in women. Models adjusted for age, diabetes, and BMI. Error bars: SE of means. The symbol “*” means interaction between MedDiet and GRS.

We also analyzed the gene–MedDiet interaction for the combined GRS including SNPs in the genes (*TERT*, *TERF2*, *RTEL1* and *DCAF4*; see Figure S2) with adherence to the MedDiet as a dichotomous variable. In a model additionally adjusted for sex, age, BMI and diabetes, no statistical significance was found for the interaction term (*p* = 0.901), indicating an additive effect. In women, the main effects for adherence to the MedDiet were higher than in men. Figure 7 shows the joint additive effects of the GRS and adherence to the MedDiet on DNAmTL in women.

The effect allele of the GRS was associated with reduced DNAmTL (resulting in −0.139 ± 0.055 kb in subjects having six or seven effect alleles in comparison with subjects having one or two effect alleles; *p* = 0.010). However, this genetic influence can be counteracted by a higher adherence to the MedDiet (associated with an increase in DNAmTL of 0.071 +/− 0.026 kb; *p* = 0.007).

Likewise, when we examined the gene–MedDiet interaction between the hit SNPs associated with DNDmTL in the GWAS (rs9529615-intergenic; see Table 5) in the same statistical model, we did not find a statistically significant interaction term (*p* = 0.994).

Furthermore, we explored at the genome-wide level the interaction term between the SNPs and adherence to the MedDiet (two categories) in the whole population. We did not find any gene–MedDiet interaction at the GWAS level of significance but obtained some interactions at the suggestive GWAS level of significance. Figure S7 shows the corresponding Manhattan plot for the statistical significance of the gene–MedDiet interactions terms in determining TL (DNAmTlAdjAge). Among the top-ranked SNPs involved in the gene–MedDiet interaction, there were some intergenic: one in chromosome 1 located in the PATJ (PATJ Crumbs Cell Polarity Complex Component) gene (rs11590158) and another in chromosome 5 located in the *ADMTS16* (thrombospondin motifs 16) gene (rs16875241). These gene–diet interactions must be further characterized through additional research employing larger sample sizes.

## 4. Discussion

To date, one of the most often used biomarkers of aging in epidemiological studies examining risk factors for chronic diseases has been TL [21,30,31,32,33,34,35,36]. However, methodological limitations exist for measuring them in large-scale studies [43,44,45]. In this investigation, we have analyzed the association between adherence to the MedDiet and TL using the new method based on the methylation algorithms published by Lu et al. [46]. To our knowledge, this is the first study analyzing the relationship between adherence to the MedDiet and TL utilizing the DNAmTL estimator. Similarly, this research represents the first investigation of the association between variants at the genome-wide level and DNAmTL in a Mediterranean Spanish population. A few GWAS studies have been published on DNAmTL, such as an investigation including European Americans and African Americans [51], as well as a study focusing on participants recruited in Canada [109]. Considering that linkage disequilibrium and other population characteristics may affect the specific variants most significantly associated in GWASs, it is necessary to conduct studies on multiple populations to assess the level of consistency or heterogeneity in the findings [118]. Similar to the detection of genetic heterogeneity in GWASs, there is also an observed heterogeneity in DNA methylation research based on the ethnic background of the participants [119,120]. Hence, despite the validation and utilization of the DNAmTL estimator in many populations [46,47,48,49,50,51,121,122,123], it is important to obtain specific information related to the Spanish population. This study represents one of the first efforts in this regard. Even while we were unable to measure TL directly in our study, we did use several indicators to test the validity of the estimator DNAmTL in the analyzed subjects. We observed statistically significant correlations between DNAmTL and biological age in the whole population as well as in separate analyses conducted for both men and women. The correlations were relatively high and as expected according to the initial validation study for this estimator [46] using the “gold standard” method of the terminal restriction fragments measured by Southern blotting [44,45,46]. In their study, Lu et al. [46] found that the utilization of DNAmTL for TL assessment showed a stronger association with biological age compared to alternative approaches like qPCR [46]. This can be related to the high coefficient of variation (CV) reported for qPCR and related measurements in previous studies [44,45,123,124,125]. Similarly, in the current study, we observed statistically significant differences in TL according to sex. Specifically, women exhibited longer DNAmTL compared to men, which was expected for this estimator [46]. Another parameter that was assessed during our initial validation process was the IAEE for the Hannum epigenetic clock [103]. This biomarker quantifies the intrinsic epigenetic age acceleration and has shown associations with the incidence of chronic diseases and mortality [99,100,101]. In our population, a statistically significant inverse association was obtained between the estimated DNAmTL and the IAEE biomarker, reinforcing the validity. In addition, we conducted an analysis to examine the relationship between SNPs in candidate genes that have been previously linked to telomere length (*TERT*, *TERF2*, *RTEL1*, *DCAF4*, *POT1*, etc.) [109] with DNAmTL. Our findings revealed numerous anticipated statistically significant associations in the expected direction, increasing consistency. In general, despite the limitation of not being able to directly quantify TL in our population, it can be stated that the new biomarker of DNAmTL acts as a reliable estimator of TL in this Mediterranean population, as previously reported by researchers in other populations [46,50,51].

Telomere attrition is a phenomenon that arises from cellular replication and is expedited by diverse environmental conditions, including inflammation and oxidative stress [126,127,128]. Oxidative stress has been recognized as a physiological factor contributing to telomere shortening, which in turn is associated with human aging by several mechanisms [127,128,129,130,131,132,133,134]. Overall, it has been suggested that the repair of oxidative damage in telomeric DNA is comparatively less efficient than in other chromosome regions [126,135]. Additionally, the presence of antioxidants has been found to slow down the loss of telomeres associated with oxidative stress [136,137]. Further investigation in human subjects is necessary to better understand the specific mechanisms involved in vivo [137]. In general, it has been accepted that the process of telomere shortening can be lowered through the adoption of lifestyle choices that are linked to decreased levels of oxidative stress [126,135,138,139]. Focusing on diet, several studies have revealed an association between a healthy food pattern and longer telomeres [65,71,140]. The MedDiet has been reported to stimulate telomerase activity in peripheral blood mononuclear cells, and if combined with moderate exercise, this diet can improve endothelial microvascular and cardiorespiratory functions, which is important for both better health and increased life expectancy [141]. In the present investigation, we have found a statistically significant association between higher adherence to the MedDiet pattern and increased DNAmTL. The observed association showed a greater magnitude in women than men. Although this is the first study to analyze the association between the MedDiet and TL using the methylation algorithm of Lu et al. [46], prior research has already been conducted investigating the relationship between adherence to the MedDiet and TL using other measuring approaches (qPCR, qFISH, or terminal restriction fragment analysis) [54,60,61,63,64,67,70,71,72,73,74,75].

The findings from prior research examining the association between adherence to the MedDiet and LT have displayed notable variability. Multiple factors have played a role in this observation, encompassing the heterogeneity of the populations under examination, differences in the questionnaires employed to assess adherence to the MedDiet, and diverse methodologies employed for measuring telomere length as well as the study design [54,60,61,63,64,67,70,71,72,73,74,75,142]. Multiple studies conducted in populations outside of the Mediterranean region, such as a multiethnic elderly population [60], older Australian men and women [64], Chinese older men and women [69], or a middle-aged population in West Virginia [65], did not find any significant associations between the derived MedDiet pattern and TL. Nevertheless, a meta-analysis [72] that synthesized the findings from multiple studies concluded that a significant association exists between higher adherence to the MedDiet and increased TL, which was primarily observed in women. Our sex-specific analysis confirmed the stronger association between adherence to the MedDiet and TL in women compared to men. The magnitude of this sex difference was more pronounced when analyzing TL as a continuous variable rather than categorizing it into two categories based on shorter and longer TL. More studies are needed to better understand the factors determining these differences. Although in our case, the number of women studied (*n* = 228) is greater than that of men (*n* = 186), conferring a greater statistical power in the stratified analyses, it is rather modest, and additional factors must be considered in order to fully account for the observed effects. Previous studies examined men and women jointly [54,60,64,65] or included only women [61], so comparisons are difficult. Furthermore, our study focused on post-menopausal women and older men, thereby limiting our ability to generalize these findings to premenopausal women and young men. Nevertheless, in the National Health and Nutrition Examination Survey (NHANES) carried out in the United States, including data on 4758 men and women aged 20–65 years [142], the statistically significant positive association between the MedDiet score and TL was only observed in women, adding more evidence to the potential extension of the sex-specific differences to younger subjects. However, in a recent study undertaken in the UK Biobank participants [75], a significant direct association between adherence to the MedDiet and TL was observed both in men and women. Hence, there is a need for more targeted investigations that specifically examine the potential disparities between sexes in relation to this association.

Although it is important to take into account that the MedDiet pattern has been noted for its advantageous synergistic effect of the overall dietary pattern, wherein the combined impact of its individual components surpasses their individual contributions [11,60], some studies have specifically investigated the foods that exhibit the strongest association with TL in addition to conducting an overall analysis of the MedDiet effects. In this regard, the results exhibit a greater degree of heterogeneity than the global pattern analysis. Thus, in the Nurses’ Health Study analyzing 4676 women [61], although the authors observed that greater adherence to the MedDiet was associated with longer telomeres, upon further examination of specific food items, they concluded that none of the individual components showed a significant association with TL [61]. In our study, when we examined the specific components of the MedDiet, we observed four remarkable statistically significant associations in women. The relevant food items were fruits, fish consumption, “sofrito” and whole grains. For all of them, compliance with the adherence to the MedDiet for the specific servings was associated with statistically longer TL in women. In men, some of these components tended to be significant or were significant, such as whole grains. Moreover, we created a new score of adherence to these four items of the MedDiet global score, and we obtained statistically significant associations with TL for the whole population and even for men in the statistical model adjusted for age, diabetes and BMI. These findings suggest an increased role of these particular components in the beneficial effects of the MedDiet on TL in both men and women. Previous research examining these particular food items supports our findings [62,71,143,144,145,146,147,148,149,150]. Fruits are known for their substantial content of dietary antioxidant chemicals and fiber, which have been linked to a greater TL in the NHANES study [143]. Fish is a dietary source abundant in omega-3 fatty acids, which have been linked to longer TL in some investigations [147]. Whole grains are known for their high content of dietary fiber, which has been associated with longer telomeres in many studies, including the NHANES study [148] and other research [71,145]. Sofrito, a key component of the MedDiet, is a mix of tomato, onion, garlic, and olive oil, which contains phenolic compounds and carotenoids [149]. It has been demonstrated that sofrito can inhibit oxidative stress [150], contributing to the favorable observed effects.

One strength of our study is the use of a carefully validated score to assess adherence to the MedDiet [93]. Additionally, our study participants reside in a region where the MedDiet is well characterized and there is favorable accessibility to such foods. These characteristics contribute to facilitating good compliance with the global MedDiet score and its individual components among participants who have a preference for it compared to countries outside of the Mediterranean region. While longevity is related to a healthy diet, it is also influenced by a whole range of other factors such as genetics. Thus, another strength of our work compared to the previously published studies on the effects of the MedDiet on TL is that in the present investigation, we have incorporated a comprehensive measurement of genetic factors. None of the previous studies, whether observational or intervention with the MedDiet [54,60,61,63,64,67,70,71,72,73,74,75,142,151], has carried out a multigenic analysis of candidate genes for TL or of GWAS to know the possible influence of the same on the TL. In our study, we have obtained some statistically significant associations between SNPs in candidate genes for TL previously discovered in GWAS [109] in the expected direction (increasing or decreasing TL). We also built a GRS with SNPs in the *TERT*, *TERF2*, *RTEL1*, and *DCAF4* candidate genes and determined the cumulative effect on DNAmTL and the MedDiet–GRS interactions. No multiplicative interaction term was observed, but the MedDiet and GRS had additive effects, suggesting a biological modulation [152]. Finally, we conducted an exploratory GWAS to better understand the SNPs and genes associated with DNAmTL. The vast majority of previous GWASs have been carried out using the TL variable measured by various techniques [82,83,84,85,86,89,90,109,153] but without using the DNAmTL indicator. Although by examining SNPs in TL-associated candidate genes, we have obtained significant associations with *TERT*, *TERF1*, *RTEL1* and *DCAF4*, the statistical significance of these associations has not been very high. This could be because one limitation of our study is the small sample size compared to the large GWAS meta-analysis. However, another factor that could influence is that the DNAmTL indicator measured additional information to that provided by the classical methods for measuring TL (qPCR, qFish, etc.). This possibility has already been proposed by Lu et al. [46], indicating that DNAmTL and TL may have different patterns of SNP associations. Indeed, in conducting the GWAS in this population, we have found the most significant associations with the suggesting level of association of GWAS (*p* < 1 × 10^−5^) with some SNPs other than the TL candidates. The hit has been intergenic on chromosome 13 and has not been previously reported. In addition, we found a very interesting signal for several SNPs on chromosome 7, including the *NCAPG2* gene. This gene plays an essential role in chromosome condensation and segregation during mitosis [154]. It has also been involved in DNA damage repair/DNA replication/telomere maintenance in functional studies [155] but has not been identified in previous GWAS. This intriguing gene scored 8 for SNP rs1466210 in the FORGEdb [111,112], suggesting potential functionality. Another top-ranked SNP in the exploratory GWAS was observed in the *ABI3BP* gene. This gene holds significant interest due to the limited number of prior research that has undertaken GWAS for DNAmTL [51,87]. One of the studies was conducted with 107 individuals, resulting in limited statistical power [87]. The second study was conducted using a sample of 297 individuals of European–American descent and 280 individuals of African–American descent [51]. The *ABI3BP* gene emerged as one of the highest-ranked genes in the GWAS conducted on African Americans. Hence, we consider our discovery to be an outcome of replication. The *ABI3BP* gene, encoding mediators of senescence, has previously been implicated in cancer, immunity, and cardiovascular risk [156,157]. However, it has not yet been identified in GWAS for TL. Therefore, additional research with larger sample sizes is necessary to replicate and discover SNPs associated with DNAmTL. Although our study has significant strengths mentioned above, it also has limitations related to the sample size and population characteristics. This investigation has been carried out in an elderly and high-risk cardiovascular population; therefore, the results cannot be extrapolated directly to younger age and more healthy populations. Specific studies of the association between adherence to MedDiet and DNAmTL would need to be carried out in these individuals. Moreover, it is important to conduct further investigations of the sex-specific effects as well as of the gene–MedDiet interactions on TL in diverse populations with enhanced statistical power.

## Figures and Tables

**Table 1:** Demographic, clinical and lifestyle characteristics of the study population according to sex.

	Total (*n* = 414)	Men (*n* = 186)	Women (*n* = 228)	*p*
Age at recruitment ^1^ (years)	65.1 ± 0.2	63.8 ± 0.4	66.1 ± 0.3	<0.001
Age at the baseline visit ^2^ (years)	65.7 ± 0.2	64.4 ± 0.4	66.7 ± 0.3	<0.001
Weight (Kg)	84.3 ± 0.7	92.3 ± 1.0	77.8 ± 0.7	<0.001
BMI (Kg/m^2^)	32.3 ± 0.2	32.2 ± 0.3	32.4 ± 0.2	0.440
Waist circumference (cm)	105.9 ± 0.5	110.9 ± 0.6	101.8 ± 0.6	<0.001
SBP (mm Hg)	141.9 ± 0.9	143.7 ± 1.4	140.5 ± 1.2	0.076
DBP (mm Hg)	81.0 ± 0.5	82.6 ± 0.7	79.6 ± 0.6	0.002
Total cholesterol (mg/dL)	195.7 ± 1.8	188.1 ± 2.8	202.0 ± 2.3	<0.001
LDL-C (mg/dL)	124.3 ± 1.5	121.5 ± 2.4	126.6 ± 1.9	0.096
HDL-C (mg/dL)	51.6 ± 0.6	47.3 ± 0.8	55.1 ± 0.7	<0.001
Triglycerides ^3^ (mg/dL)	141.3 ± 2.9	139.0 ± 4.0	143.1 ± 4.2	0.488
Fasting glucose (mg/dL)	113.4 ± 1.4	113.7 ± 2.2	113.2 ± 1.8	0.875
Physical activity ^4^ (MET·min/wk)	1708 ± 78	1941 ± 133	1519 ± 89	0.007
Adherence to MedDiet (17-I) ^5^	8.0 ± 2.8	7.9 ± 2.8	8.1 ± 2.7	0.210
High adherence to MedDiet ^6^ (≥9) n (%)	172 (41.5)	75 (40.3)	97 (42.5)	0.648
Educational level				<0.001
Primary n (%)	255 (61.6)	92 (49.5)	163 (71.5)	<0.05
Secondary n (%)	87 (21.0)	55 (29.6)	32 (14.0)	<0.05
University n (%)	72 (17.4)	39 (21.0)	33 (14.5)	<0.05
Current smokers n (%)	48 (11.6)	30 (16.1)	18 (7.9)	<0.001
Never smokers n (%)	188 (45.4)	32 (17.2)	156 (68.4)	<0.001
Former smokers n (%)	178 (43.0)	124 (66.7)	54 (23.6)	<0.001
Type 2 diabetes n (%)	164 (39.6)	75 (40.3)	89 (39.0)	0.790
On metformin n (%)	128 (30.9)	60 (32.3)	68 (29.8)	0.594
On insulin n (%)	21 (5.1)	11 (5.9)	10 (4.4)	0.481
Lipid-lowering drugs n (%)	274 (66.2)	125 (67.2)	149 (65.4)	0.692
Blood pressure medication n (%)	327 (79.0)	149 (80.1)	178 (78.1)	0.613

Values are mean ± SE for continuous variables and number (%) for categorical variables. BMI: body mass index; SBP: systolic blood pressure; DBP: diastolic blood pressure; LDL-C: low-density lipoprotein cholesterol; HDL-C: high-density lipoprotein cholesterol; *p*: *p*-value for the comparisons (means or %) between men and women. Student’s t test was used to compare means and Chi-squared tests were used to compare categories. ^1^ Age in years at recruitment according to the inclusion criteria for age. ^2^ Age in years at the baseline visit when the blood sample for methylation analysis was drawn. ^3^ Triglycerides was ln-transformed for statistical testing. ^4^ Total physical activity was computed. ^5^ Quantitative 17-item (17-I) questionnaire for adherence to the Mediterranean diet (MedDiet). ^6^ High adherence to MedDiet ≥ 9 points in the 17-I score.

**Table 2:** Leukocyte telomere length–DNA methylation (DNAmTL), DNAmTL adjusted for age (DNAmTLAdjAge) and intrinsic epigenetic age acceleration (IEAA), based on Hannum’s calculation, per sex.

	Total (*n* = 414)	Men (*n* = 186)	Women (*n* = 228)	*p*
DNAmTL (Kb)	6.6958 ± 0.0107	6.6693 ± 0.0172	6.7174 ± 0.0133	0.025
DNAmTLAdjAge ^1^ (Kb/year)	−0.0018 ± 0.0101	−0.0431 ± 0.0162	0.0321 ± 0.0122	<0.001
Subjects with shorter TL ^2^, *n* (%)	194 (46.9)	100 (53.8)	94 (41.2)	0.011
IEAA Hannum ^3^	−0.0003 ± 0.238	0.6540 ± 0.3567	−0.5364 ± 0.3153	0.013

Values are mean ± SE for continuous variables and number (%) for categorical variables. DNAmTL: telomere length–DNA methylation; Kb: kilobases; *p*: *p*-value for the comparisons (means or %) between men and women. TL: telomere length. ^1^ DNAmTL adjusted for age. ^2^ Subjects with shorter telomere length having negative values for the computed DNAmTLAdjAge. ^3^ Intrinsic epigenetic age acceleration (IEAA) based on Hannum’s calculation.

**Table 3:** Association between the 10 most significant (top-ranked) methylation sites included in the leukocyte DNAmTL computation algorithm and the estimated DNAmTL in this population.

CpG Site ^1^	Gene Symbol	Chr	BP	*p* ^2^	*r* ^3^
cg21566642	*NCAPG*	4	17813558	5.80 × 10^−20^	0.43869
cg14391737	*CCND3*	6	41904398	2.25 × 10^−16^	0.39760
cg01940273	*TPI1*	12	6977747	1.92 × 10^−15^	0.38594
cg17739917	*DHRS3*	1	12664243	4.70 × 10^−15^	0.38093
cg21911711	*DNTT*	10	98062687	1.81 × 10^−12^	−0.34522
cg00475490	*YPEL3*	16	30106682	1.02 × 10^−10^	−0.31819
cg24859433	*TPST1*	7	65817282	4.23 × 10^−9^	0.29048
cg18110140	*CCDC102B*	18	66389420	4.82 × 10^−9^	0.28946
cg09935388	*SRRM3*	7	75897850	7.19 × 10^−9^	0.28630
cg25648203	*C7orf41*	7	30185776	9.24 × 10^−9^	−0.28430

Chr: chromosome; BP: base position in the chromosome (Homo Sapiens GRCh37.p13 genome build). ^1^ Supplementary Table S2 shows a more complete list of the methylation sites ordered by *p*-value in this population and also the direct or inverse effect in each site in the original work of Lu et al., 2019 [46]. ^2^ *p*-value obtained in this population between the corresponding CpG site and the estimated global DNAmTL (*n* = 414). Models were adjusted for sex, age, diabetes, body mass index, batch effect and leukocyte cell-types. ^3^ Partial correlation coefficient in the multivariate adjusted model (described above) for the corresponding methylation site and the global DNAmTL.

**Table 4:** Association between SNPs in candidate genes for telomere length reported in the literature and the DNAmTL in this population.

Gene Symbol	Chr	SNP	BP	MAF	Beta	*p* ^1^
*TERT*	5	rs2075786	1266310	0.326	−0.0407	0.0078
*TERF2*	16	rs3785073	69401937	0.238	−0.0424	0.0086
*TERF2*	16	rs3743669	69398353	0.197	−0.0390	0.0179
*RTEL1*	20	rs6011011	62299578	0.140	0.0551	0.0193
*TERT*	5	rs2736100	1286516	0.472	−0.0328	0.0274
*TERF2*	16	rs9939870	69396585	0.315	−0.0356	0.0277
*RTEL1*	20	rs6011002	62297802	0.092	0.0498	0.0375
*DCAF4*	14	rs17781739	73392839	0.182	−0.0309	0.0376
*RTEL1*	20	rs3848668	62293272	0.055	0.0513	0.0390
*DCAF4*	14	rs12885397	73408134	0.294	0.0324	0.0545
*POT1*	7	rs17246404	124462661	0.222	−0.0288	0.0677
*SENP7*	3	rs9870022	101071429	0.215	−0.0346	0.0696
*ATM*	11	rs3218674	108115587	0.012	0.1189	0.0696
*OBFC1*	10	rs3814219	105647095	0.199	0.0282	0.0739
*PARP1*	1	rs8679	226548554	0.108	0.0297	0.0780
*PARP1*	1	rs2271347	226549498	0.108	0.0293	0.0804
*SENP7*	3	rs6772703	101232736	0.282	0.0267	0.0831
*TERF2*	16	rs251796	69395434	0.196	0.0274	0.0942
*ATM*	11	rs1800058	108160350	0.022	0.0900	0.0951
*ZNF208*	19	rs12608935	22145147	0.351	−0.0236	0.0986

Chr: chromosome; SNP: single nucleotide polymorphism. BP: base position in the chromosome (Homo Sapiens GRCh37.p13 genome build); MAF: minor allele frequency; Beta: regression coefficient indicates the effect for the minor allele on DNAmTL. Models adjusted for sex and age. ^1^ *p*-value obtained in the regression models adjusted for sex and age for each SNP using a genetic additive approach (*n* = 414).

**Table 5:** Association between adherence to the Mediterranean diet (MedDiet) and shorter leukocyte telomere length ^1^ in the whole population and by sex.

	Total (*n* = 414) OR (95% CI)	*p*	Men (*n* = 186) OR (95% CI)	*p*	Women (*n* = 228) OR (95% CI)	*p*
Adherence to MedDiet (17-I) ^2^						
Model 1 ^3^	0.92 (0.86–0.99)	0.032	0.96 (0.87–1.07)	0.444	0.89 (0.81–0.98)	0.028
Model 2 ^4^	0.93 (0.86–0.99)	0.034	0.96 (0.87–1.07)	0.470	0.89 (0.81–0.99)	0.031
Model 3 ^5^	0.93 (0.85–0.99)	0.049	0.99 (0.86–1.11)	0.904	0.88 (0.79–0.98)	0.023

OR: odds ratio, indicates the effect for the minor allele on adherence to the Mediterranean diet (MedDiet) and telomere length; CI: confidence interval. Values are OR and 95% CI of having shorter telomere length for a 1 point increase in adherence to MedDiet. ^1^ Subjects with shorter telomere length are those subjects having negative values for the computed DNAmTLAdjAge (*n* = 194) (see Table 2). ^2^ Quantitative 17-item (17-I) questionnaire for adherence to MedDiet. ^3^ Model 1 adjusted for sex and age. ^4^ Model 2 adjusted for sex, age, diabetes, and body mass index (BMI). ^5^ Model 3 adjusted for sex, age, diabetes, BMI, metformin, insulin, lipid-lowering drugs, hypertension medication, systolic blood pressure, education, smoking, and physical activity.

**Table 6:** Association between adherence to the Mediterranean diet (MedDiet) and telomere length (DNAmTL) by sex, considering the whole pattern and the score of the 4 MedDiet items (fruit, fish, “sofrito” and whole grains).

	Total (*n* = 414)	*p*	Men (*n* = 186)	*p*	Women (*n* = 228)	*p*
	β (SE)		β (SE)		β (SE)	
Adherence to MedDiet (17-I) ^1^						
Model 1 ^2^	0.006 (0.004)	0.095	−0.003 (0.006)	0.588	0.014 (0.005)	0.002
Model 2 ^3^	0.006 (0.004)	0.113	−0.003 (0.006)	0.648	0.014 (0.005)	0.003
Model 3 ^4^	0.005 (0.004)	0.147	−0.006 (0.006)	0.332	0.015 (0.005)	0.003
High adherence to MedDiet (2 categories) ^5^						
Model 1 ^2^	0.042 (0.020)	0.041	0.001 (0.033)	0.994	0.077 (0.025)	0.003
Model 2 ^3^	0.040 (0.020)	0.048	0.003 (0.034)	0.922	0.075 (0.026)	0.004
Model 3 ^4^	0.036 (0.021)	0.080	−0.016 (0.034)	0.637	0.078 (0.026)	0.004
Score of 4 MedDiet items ^6^						
Model 1 ^2^	−0.237 (0.055)	<0.001	−0.204 (0.103)	0.048	−0.295 (0.065)	<0.001
Model 2 ^3^	−0.233 (0.055)	<0.001	−0.207 (0.104)	0.047	−0.290 (0.066)	<0.001
Model 3 ^4^	−0.217 (0.055)	<0.001	−0.133 (0.105)	0.210	−0.289 (0.067)	<0.001

Values are regression coefficients (expressed as kb per unit of adherence variable) and SEs. ^1^ Quantitative 17-item (17-I) questionnaire for adherence to Mediterranean diet (MedDiet). ^2^ Model adjusted for sex and age. ^3^ Model adjusted for sex, age, diabetes, and body mass index (BMI). ^4^ Model adjusted for sex, age, diabetes, BMI, metformin, insulin, lipid-lowering drugs, hypertension medication, systolic blood pressure (SBP), education, smoking, and physical activity. ^5^ High adherence to MedDiet ≥ 9 points in the 17-I score compared with low adherence. ^6^ Score 4: The 4 MedDiet items (fruit, fish, “sofrito” and whole grains) most significantly associated with TL in women (see Supplemental Material). Estimators comparing category 1 (zero adherence) with category 5 (0 to 4 points) are shown.

**Table 7:** Top-ranked SNPs in the GWAS for DNAmTL in the whole population. Model adjusted for sex and age.

Chr	SNP	BP	Beta	*p* ^1^	MAF	Gene Symbol	FORGEdb Score
13	rs9529615	35307066	0.07256	2.33 × 10^−7^	0.32708	intergenic	5
7	rs2178528	158393488	−0.07369	8.30 × 10^−7^	0.24521	intergenic	6
3	rs662538	13135500	−0.13220	1.18 × 10^−6^	0.02236	*IQSEC1*	8
7	rs11983468	158412279	−0.07504	1.86 × 10^−6^	0.19868	intergenic	7
7	rs11763040	158416203	−0.07571	2.06 × 10^−6^	0.20367	intergenic	8
7	rs1466210	158444975	−0.07824	4.78 × 10^−6^	0.19848	*NCAPG2*	8
7	rs7788516	158410592	−0.07127	5.85 × 10^−6^	0.20667	intergenic	8
1	rs1327126	115973920	0.06324	6.27 × 10^−6^	0.39677	intergenic	6
3	rs7613610	100499321	0.08068	8.09 × 10^−6^	0.27756	*ABI3BP*	7
13	rs12427518	53519692	0.13080	9.12 × 10^−6^	0.22903	intergenic	10
13	rs17504027	30587544	0.14460	1.15 × 10^−5^	0.03015	*LICO00544*	4
16	rs11076786	3811596	0.10400	1.16 × 10^−5^	0.14018	*CREBBP*	8
15	rs4777407	71826919	−0.06769	1.37 × 10^−5^	0.35144	*THSD4*	4
15	rs4906654	26274113	0.09824	1.42 × 10^−5^	0.25240	*LOC100128714*	6
8	rs256	19811967	0.08308	1.42 × 10^−5^	0.14417	*LPL*	6
8	rs7837548	61802242	0.07462	1.89 × 10^−5^	0.27736	intergenic	5
1	rs11164337	102389733	−0.07336	2.01 × 10^−5^	0.46765	*OLFM3*	4

Chr: chromosome; SNP: single nucleotide polymorphism. BP: base position in the chromosome (Homo Sapiens GRCh37.p13 genome build). Beta: regression coefficient indicates the effect for the minor allele on DNAmTL; MAF: minor allele frequency. ^1^ *p* adjusted for sex and age for each SNP using a genetic additive approach.

## Data Availability

Neither the participants’ consent forms nor ethics approval included permission for open access. However, we follow a controlled data-sharing collaboration model, and data for collaborations will be available upon request pending application and approval. Investigators who are interested in this study can contact the corresponding author.

## Supplementary Materials

The following supporting information can be downloaded at https://www.mdpi.com/article/10.3390/antiox12112004/s1, Table S1: Quantitative 17-item questionnaire for adherence to Mediterranean diet; Figure S1: Association between telomere length–DNA methylation and chronological age in men (A) and in women (B); Table S2: Association between the methylation sites included in the leukocyte DNAmTL computation and the estimated DNAmTL in the population; Figure S2: Telomere length (DNAmTLAdjustAge) depending on the categories of the genetic risk score (GRS) combining the effect alleles (associated with shorter TL) for the SNPs; Table S3: Compliance (%) of adherence to the Mediterranean diet for each item of the 17-I score by sex; Table S4: Association between the particular items (foods/beverages) of the Mediterranean diet score (17-I) with telomere length by sex; Figure S3: Q-Q plot for the genome-wide association study (GWAS) of telomere length–DNA methylation adjusted for sex and age; Figure S4: Zoom plots of the selected top-ranked SNPs for the GWAS of DNAmTL; Table S5: FORGEdb total and detailed scores for the 10 top-ranked SNPs in the GWAS for DNAmTL; Figure S5: Gene expression heatmap based on the dataset GTExV8 (54 tissue types) for average gene expression of genes selected in the GWAS for DNAmTL; Table S6: Coefficients obtained in the SNP-based genome-wide association study (GWAS) for DNAmTL adjusted for additional covariates; Figure S6: Manhattan plot for the gene-based GWAS analysis for DNAmTL. Figure S7: Manhattan plot of the SNP-based GWAS analyzing the interaction between the SNPs and adherence to Mediterranean diet in determining telomere length (DNAmTLAdjustAge).
