# Supplementary material for: Associations between the New DNA-Methylation-Based Telomere Length Estimator, the Mediterranean Diet and Genetics in a Spanish Population at High Cardiovascular Risk
Source: Antioxidants (Basel). 2023 Nov 15;12(11):2004. doi: 10.3390/antiox12112004 (PMC10669035; doi:10.3390/antiox12112004)
Supplement: Supplementary file 1 [file antioxidants-12-02004-s001.zip › antioxidants-2712097-supplementary.pdf]

antioxidants

MDPI

Article

Associations between the new DNA-methylation-based telomere length estimator, the Mediterranean diet, and genetics in a Spanish population at high cardiovascular risk

Oscar Coltell, Eva M Asensio, José V. Sorlí, Carolina Ortega-Azorín, Rebeca Fernández-Carrión, Eva C Pascual, Rocío Barragán, José I González, Ramon Estruch, Juan F Alzate, Alejandro Pérez-Fidalgo, Olga Portolés, Jose M Ordovas, and Dolores Corella.

SUPPLEMENTARY MATERIAL

INDEX

Table S1. Quantitative 17-item questionnaire for Adherence to Mediterranean diet. 2
Figure S1. Association between telomere length-DNA methylation and chronological age in men (A), and in women (B).. 3
Table S2. Association between the methylation sites included in the leukocyte DNAmTL computation and the estimated DNAmTL in the population. 4
Figure S2. Telomere length (DNAmTLAdjustAge) depending on the categories of the genetic risk score (GRS) combining the effect alleles (associated with shorter TL) for the SNPs. 5
Table S3. Compliance (%) of adherence to the Mediterranean diet for each item of the 17-I score by sex.........6
Table S4. Association between the particular items (foods/beverages) of the Mediterranean diet score (17-I) with telomere length by sex. 7
Figure S3. Q-Q plot for the Genome-Wide Association Study (GWAS) of telomere length (DNAmTL) adjusted for sex and age in this population. 8
Figure S4. Zoom plots of the selected top-ranked SNPs for the GWAS of DNAmTL. 9
Table S5. FORGEdb total and detailed scores for the 10 top-ranked SNPs in the GWAS for DNAmTL 10
Figure S5. Gene expression heatmap based on the dataset GTExV8 (54 tissue types), showing the average expression for the IQSEL1, NACPG2 and PTPRN2 genes selected in the GWAS for DNAmTL......................10
Table S6. Coefficients obtained in the SNP-based Genome-Wide Association Study (GWAS) for DNAmTL adjusted for additional covariates 11
Figure S6. Manhattan plot for the gene-based GWAS analysis for telomere length-DNA methylation adjusted for age 12
Figure S7. Manhattan plot of the SNP-based GWAS analizing the interaction between the SNPs and adherence to Mediterranean Diet in determining telomere length (DNAmTLAdjustAge). 12

antioxidants MDPI

Table S1. Quantitative 17-item questionnaire for Adherence to Mediterranean diet.

**Food items and frequency of consumption**	**Criteria for 1 point**[1]
1. Do you use extra virgin olive oil as the main culinary fat?	Yes
2. How many servings of vegetables do you consume per day? (Count garnish and side servings as ½ point; 1 serving =200g)	≥2
3. How many pieces of fruit or 100% natural fruit juice do you consume per day?	≥3
4. How many servings of red meat, hamburgers or meat products (ham, sausage, etc.) do you consume per week? (1 serving = 100–150g)	≤1
5. How many servings of butter, margarine, or cream do you consume per week? (1 serving = 12g)	<1
6. How many sugar-sweetened beverages (sodas, tonic waters, energy drinks, fruit juices with added sugar) do you consume per week?	<1
7. How many servings of legumes do you consume per week? (1 serving = 150g)	≥3
8. How many servings of fish / shellfish do you consume per week? (1 serving = 100-150g, or 4-5 pieces of fish, or 200g of shellfish)	≥3
9. How many times per week do you consume pastries, such as cookies, sweets or cakes?	<3
10. How many times per week do you consume nuts[2]? (1 serving = 30g)	≥3
11. Do you preferentially consume chicken, turkey, or rabbit meat instead of beef, pork, hamburgers, or sausage?	Yes
12. How many times per week do you consume vegetables, pasta, rice, or other dishes seasoned with *sofrito* (sauce made with tomato and onion, leek, or garlic and simmered with olive oil)?	≥2
13. Do you add sugar to beverages (coffee, tea)?	No/use artificial sweeteners
14. How many servings of white bread do you consume per day? (1 serving = 75g)	≤1
15. How many servings of whole grains (bread, rice, pasta) do you consume per week?	≥5
16. How many servings of refined bread, rice and/or pasta do you consume per week?	<3
17. Do you drink wine? How much do you consume per week? (1 glass = 100ml)	Men: 2-3 glasses/day<br>Women:1-2 glasses/day

[1] 0 points if the criteria is not met. [2] including peanuts.

antioxidants

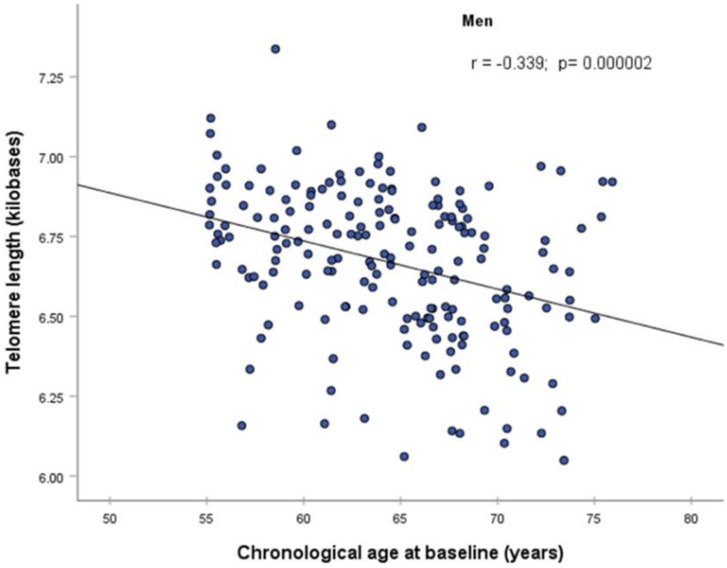

A

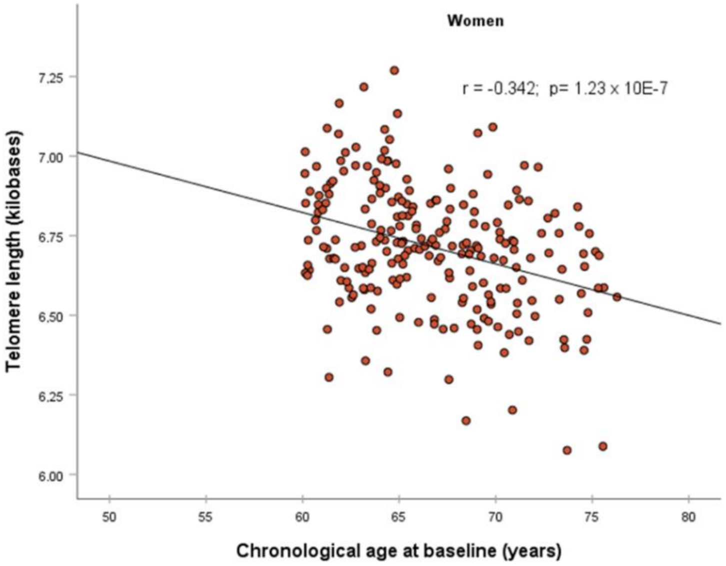

B

Figure S1. Association between telomere length-DNA methylation and chronological age in men (A), and in women (B). Scatter plots with raw values, Pearson correlation coefficients and p-values.

Table S2. Association between the methylation sites included in the leukocyte DNAmTL computation and the estimated DNAmTL in the population.

CpG site[1]	Gene Symbol	Chr	BP	*p*[2]	*r*[3]	Coefficient[4]
cg21566642	NCAPG	4	17813558	5.80E-20	0.4387	0.1155
cg14391737	CCND3	6	41904398	2.25E-16	0.3976	0.6714
cg01940273	TPI1	12	6977747	1.92E-15	0.3859	0.2302
cg17739917	DHRS3	1	12664243	4.70E-15	0.3809	0.2783
cg21911711	DNTT	10	98062687	1.81E-12	-0.3452	-0.0719
cg00475490	YPEL3	16	30106682	1.02E-10	-0.3182	-0.2502
cg24859433	TPST1	7	65817282	4.23E-09	0.2905	0.0222
cg18110140	CCDC102B	18	66389420	4.82E-09	0.2895	0.1152
cg09935388	SRRM3	7	75897850	7.19E-09	0.2863	0.2527
cg25648203	C7orf41	7	30185776	9.24E-09	-0.2843	-0.3828
cg25189904	NA-CHL1	3	153242	1.02E-08	0.2835	0.1325
cg19572487	MMEL1-ACTRT2	1	2778841	1.44E-08	0.2807	0.0549
cg26703534	FREM1	9	14910689	1.59E-08	0.2799	0.0909
cg01901332	PRMT2	21	48081242	2.99E-08	0.2747	0.2711
cg03636183	CALN1	7	71800412	6.55E-08	0.2681	0.0950
cg17287155	ELOVL2	6	11044877	8.49E-08	-0.2658	-0.9413
cg04551776	IQCE	7	2644443	1.08E-07	0.2638	0.6262
cg15342087	LIPF	10	90428384	1.26E-07	-0.2624	-0.3370
cg05086879	FAM69A	1	93416982	1.63E-07	0.2601	0.1645
cg05575921	PDCD1LG2	9	5510497	4.33E-07	0.2513	0.2854
cg23161492	BAT3	6	31607674	4.50E-07	0.2510	0.0642
cg11660018	NPTX2	7	98245716	5.94E-07	-0.2484	-0.0383
cg05284742	ANKRD11	16	89387014	7.01E-07	0.2469	0.1133
cg11556164	AMZ1-GNA12	7	2764599	8.99E-07	-0.2445	-0.0010
cg14580211	COL6A1	21	47404109	1.48E-06	0.2398	0.3663
cg16841366	TCF3	19	1617027	2.79E-06	-0.2335	-0.2229
cg19859270	TRIM8	10	104406990	3.32E-06	-0.2318	-0.2483
cg18146737	DGKI	7	137223417	4.28E-06	0.2292	0.0869
cg04535902	TARP-AMPH	7	38351226	4.92E-06	0.2278	0.0619
cg17738628	SERINC5	5	79490511	1.06E-05	-0.2199	-0.0278
cg02319782	IQSEC3-SLC6A12	12	291668	1.90E-05	0.2136	0.6466
cg07739478	C6orf25	6	31692234	2.26E-05	0.2117	0.0443
cg06132400	TARP-AMPH	7	38350938	3.64E-05	0.2064	0.0482
cg13357922	SPRN-LOC619207	10	135251512	4.16E-05	0.2049	0.1159
cg00580497	SLC12A7-SLC6A19	5	1153746	4.47E-05	0.2041	0.2946

BMI: Body Mass Index; Chr: chromosome; SNP: single nucleotide polymorphism. BP: Base position of the probe in the chromosome (Homo Sapiens GRCh37.p13 genome build). Beta: regression coefficient indicates the effect for the minor allele on DNAmTL; MAF: minor allele frequency.

[1.] Top-ranked CpGs included in the DNAmTL algorithm according to Lu et al. [46].

[2.]p-value obtained in this population between the corresponding CpG site and the estimated global DNAmTL. The model was adjusted for sex, age, diabetes, body mass index, batch effect and leukocyte cell-types.

[3.]r partial correlation coefficient of the corresponding methylation site and the estimated global DNAmTL in this population.

[4.]Coefficient from Lu, et al. [46].

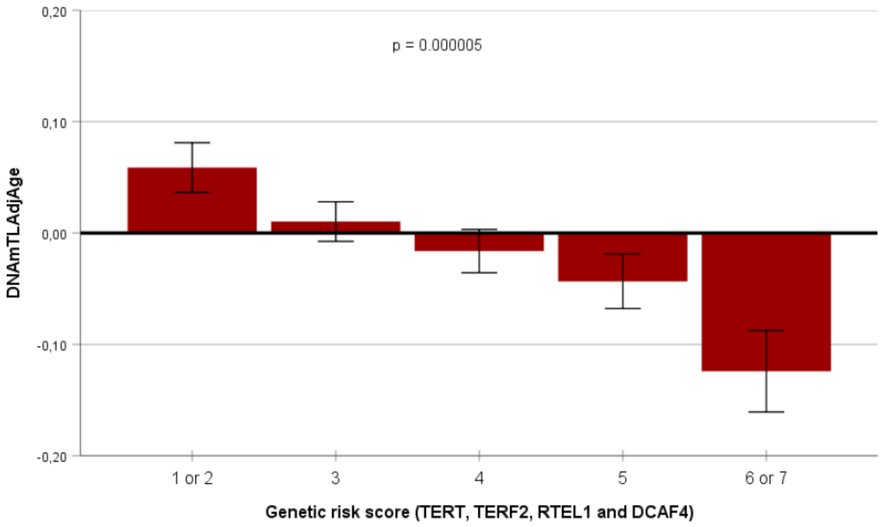

Figure S2. Telomere length (DNAmTLAdjustAge) depending on the categories of the genetic risk score (GRS) combining the effect alleles (associated with shorter TL) for the SNPs: rs2075786-TERT, rs37850-TERF2, rs6011011-RTEL1, and rs17781739-DCAF4. Scores 1 and 2, and 6 and 7 were combined. P=0.0002 for association in a model adjusted for age, sex, diabetes and body mass index. P-value obtained in the adjusted model fore the trend in the GRS score.

Table S3. Compliance (%) of adherence to the Mediterranean diet for each item of the 17-I score by sex.

Food items and frequency of consumption	Total (n=414)	Men (n=186)	Women (n=228)	*p*
1. Do you use olive oil as main culinary fat?	55.1	57.5	53.1	0.365
2. How many vegetable servings do you consume per day?	51.7	44.1	57.9	0.005
3. How many pieces of fruit or 100% natural fruit juice do you consume per day?	50.0	48.4	51.3	0.553
4. How many servings of red meat, hamburger, or meat products do you consume per week?	47.6	44.6	50.0	0.276
5. How many servings of butter, margarine, or cream do you consume per week?	82.4	81.2	83.3	0.568
6. How many sugar-sweetened beverages do you consume per week?	57.7	54.8	60.1	0.282
7. How many servings of legumes do you consume per week?	12.3	15.1	10.1	0.126
8. How many servings of fish / shellfish do you consume per week?	43.0	40.9	44.7	0.428
9. How many times per week do you consume pastries, such as cookies, sweets, or cakes?	44.7	47.8	42.1	0.242
10. How many times per week do you consume nuts[2]?	41.5	45.7	38.2	0.121
11. Do you preferentially consume chicken, turkey, or rabbit meat instead of beef, pork, hamburgers, or sausage?	77.5	73.7	80.7	0.088
12. How many times per week do you consume vegetables, pasta, rice, or other dishes seasoned with sofrito?	63.0	63.4	62.7	0.880
13. Do you add sugar to beverages (coffee, tea)?	68.4	57.0	77.6	<0.001
14. How many servings of white bread do you consume per day?	39.1	32.8	44.3	0.017
15. How many servings of whole grains do you consume per week?	27.1	20.4	32.5	0.006
16. How many servings of refined bread, rice and/or pasta do you consume per week?	25.6	28.5	23.2	0.224
17. Do you drink wine? How much do you consume per week?	17.4	29.0	7.9	<0.001

Values are % compliance (see Table S1 for compliance details) for each item of the adherence to the Mediterranean diet (MedDiet) of 17 points. p: *p*-values for differences between men and women.

Table S4. Association between the particular items (foods/beverages) of the Mediterranean diet score (17-I) with telomere length by sex. P-values for the multivariable adjusted model are shown.

Food items and frequency of consumption	Total (n=414) p	Men (n=186) p	Women (n=228) p
1. Do you use olive oil as main culinary fat?	0.802	0.504	0.274
2. How many vegetable servings do you consume per day?	0.603	0.716	0.718
3. How many pieces of fruit or 100% natural fruit juice do you consume per day?	0.009	0.184	0.014
4. How many servings of red meat, hamburger, or meat products do you consume per week?	0.862	0.328	0.192
5. How many servings of butter, margarine, or cream do you consume per week?	0.515	0.112	0.426
6. How many sugar-sweetened beverages do you consume per week?	0.803	0.087	0.134
7. How many servings of legumes do you consume per week?	0.638	0.344	0.628
8. How many servings of fish / shellfish do you consume per week?	0.165	0.855	0.010
9. How many times per week do you consume pastries, such as cookies, sweets, or cakes?	0.226	0.074	0.876
10. How many times per week do you consume nuts[2]?	0.979	0.341	0.325
11. Do you preferentially consume chicken, turkey, or rabbit meat instead of beef, pork, hamburgers, or sausage?	0.457	0.340	0.965
12. How many times per week do you consume vegetables, pasta, rice, or other dishes seasoned with sofrito?	0.031	0.731	0.005
13. Do you add sugar to beverages (coffee, tea)?	0.386	0.444	0.677
14. How many servings of white bread do you consume per day?	0.561	0.780	0.572
15. How many servings of whole grains do you consume per week?	0.003	0.042	0.011
16. How many servings of refined bread, rice and/or pasta do you consume per week?	0.345	0.408	0.014
17. Do you drink wine? How much do you consume per week?	0.791	0.844	0.860

Values are *p*-values for the association between each particular item of the score and DNAmTL by sex (models adjusted for age, sex, diabetes and BMI) for each item of the adherence to the Mediterranean diet (MedDiet) of 17 points. See criteria for compliance in Table S1.

antioxidants

MDPI

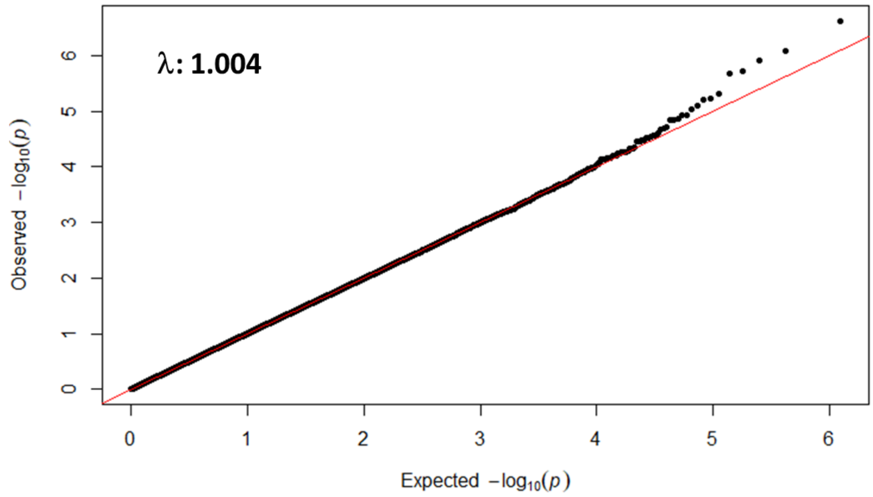

Figure S3. Q-Q plot for the Genome-Wide Association Study (GWAS) of telomere length (DNAmTL) adjusted for sex and age in this population. λ: lambda.

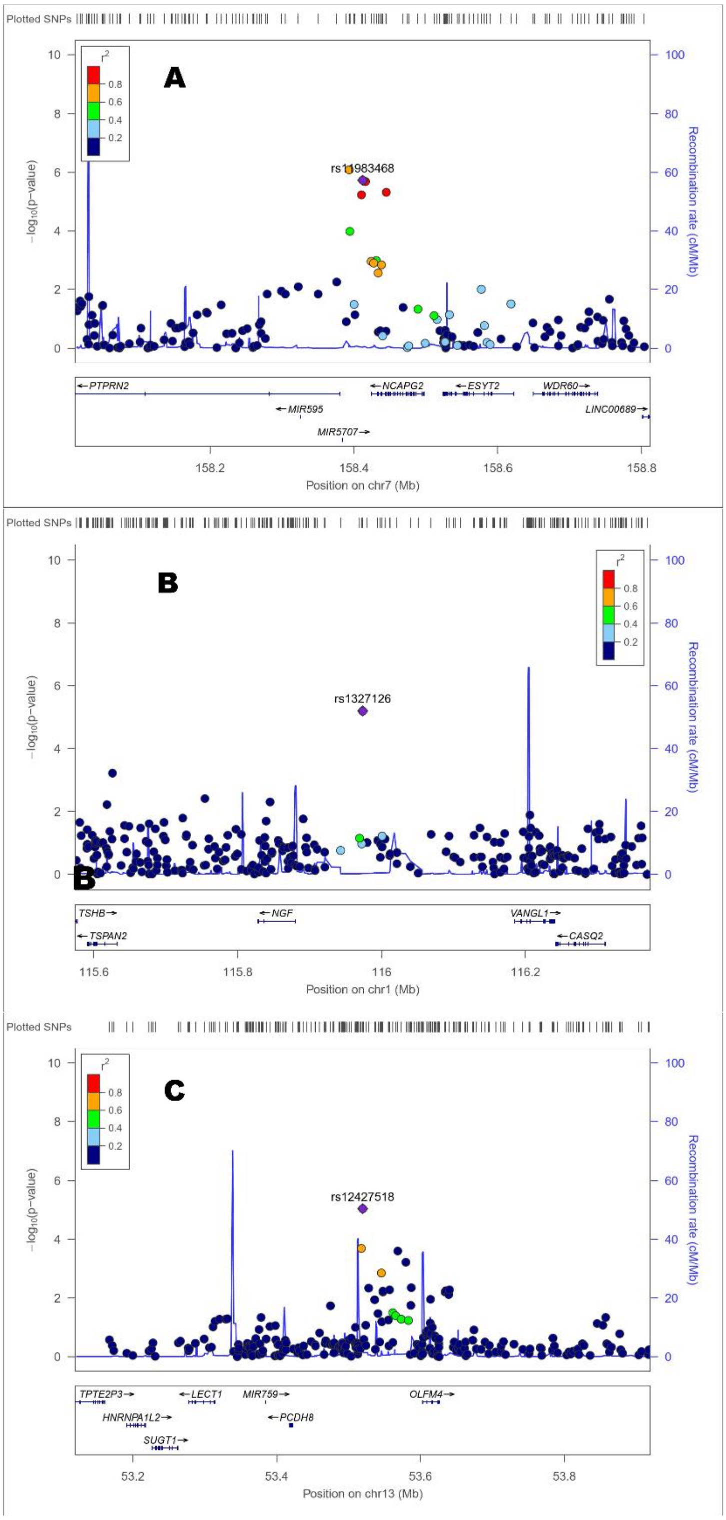

Figure S4. Zoom plots of the selected top-ranked SNPs for the GWAS of DNAmTL in this population (Table 5). The panel shows the following SNPs: A) rs11983468 (Chr.: 7; *p*=1.86E-06); B) rs1327126 (Chr.: 1; *p*=6.27E-06); C) rs12427518 (Chr.: 13; *p*=9.12E-06).

Table S5. FORGEdb total and detailed scores for the 10 top-ranked SNPs in the GWAS for DNAmTL in the whole population (Table 5).

	rs9529615	rs2178528	rs662538	rs11983468	rs11763040	rs1466210	rs7788516	rs1327126	rs7613610	rs12427518
Summary and number of annotations for each SNP (FORGEdb score)	**5**	**6**	**8**	**7**	**8**	**8**	**8**	**6**	**7**	**10**
RefSeq closest gene data	1	1	1	1	1	1	1	1	1	1
UCSC genome browser data	1	1	1	1	1	1	1	1	1	1
CADD v1.6 annotations	3	3	3	3	3	3	3	3	3	3
GTEx cis-eQTLs	0	7	0	11	12	19	9	1	4	0
eQTLGen blood cis-eQTLs	0	3	1	3	3	3	3	0	3	2
ABC contacts	0	0	0	0	0	0	10	0	0	1
FORGE2-TF motifs	2	0	12	0	3	1	0	0	0	2
CATO score	0	0	1	1	1	1	0	0	1	1
FORGE2 consolidated roadmap DNase I hotspots (erc2-DHS)	0	0	5	0	0	4	6	0	1	1
FORGE2 consolidated roadmap H3 histone marks (erc2-H3-all)	45	22	64	52	47	59	60	25	20	75
FORGE2 unconsolidated roadmap DNase I hotspots (erc)	35	1	34	3	0	67	72	4	26	5
FORGE2 blueprint DNase I hotspots (blueprint)	0	0	1	0	0	0	0	0	0	18
FORGE2 ENCODE DNase I hotspots (encode)	4	7	48	6	11	30	38	11	4	3
FORGE2 consolidated roadmap chromatin states (erc2-15state)	127	127	127	127	127	127	127	127	127	127

A Forgedb score of 10 (the highest score) is computed from the presence of: 10 = eQTL + ABC + TF motif + CATO + DNase I hotspot + histone mark ChIP-seq. A FORGEdb score of 9 (the second highest score) is computed from the presence of: 9 = eQTL + ABC + {TF motif|CATO} + DNase I hotspot + histone mark ChIP-seq (Source: https://forge2.altiusinstitute.org/files/FORGEdb_scores.html).

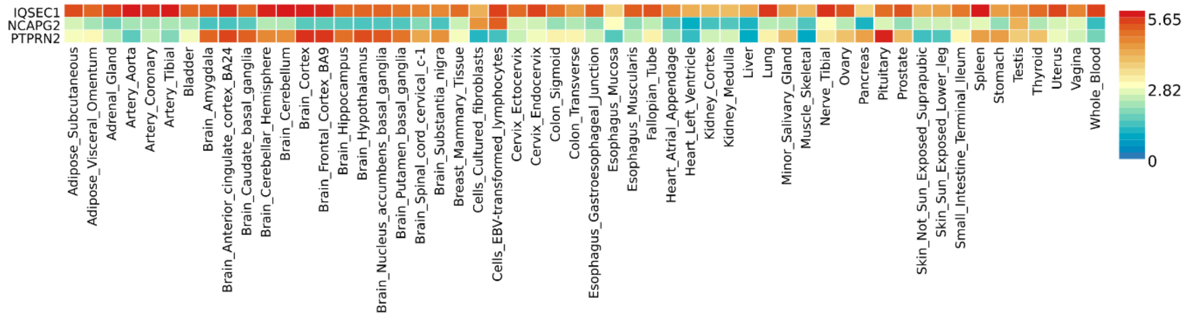

Figure S5. Gene expression heatmap based on the dataset GTExV8 (54 tissue types), showing the average expression for the IQSEL1, NACPG2 and PTPRN2 genes selected in the GWAS for DNAmTL.

Table S6. Coefficients obtained in the SNP-based Genome-Wide Association Study (GWAS) for DNAmTL adjusted for additional covariates.

Chr	SNP	BP	Beta	p[1]	MAF	Gene Symbol
13	rs9529615	35307066	0.07276	2.52E-07	0.327077	intergenic
7	rs2178528	158393488	-0.07343	9.94E-07	0.245208	intergenic
3	rs662538	13135500	-0.13040	1.81E-06	0.0223642	IQSEC1
7	rs11983468	158412279	-0.07387	3.31E-06	0.198682	intergenic
7	rs11763040	158416203	-0.07461	3.56E-06	0.203674	intergenic
7	rs1466210	158444975	-0.07691	8.24E-06	0.198482	NCAPG2
7	rs7788516	158410592	-0.07035	8.95E-06	0.206669	intergenic
1	rs1327126	115973920	0.06237	9.93E-06	0.396765	intergenic
15	rs4906654	26274113	0.10020	9.96E-06	0.252396	LOC100128714
13	rs12427518	53519692	0.13010	1.04E-05	0.229034	intergenic
3	rs7613610	100499321	0.08015	1.04E-05	0.277556	ABI3BP
16	rs11076786	3811596	0.10330	1.33E-05	0.140176	CREBBP
1	rs11164337	102389733	-0.0748	1.41E-05	0.467652	OLFM3
13	rs17504027	30587544	0.14260	1.59E-05	0.0301518	intergenic
8	rs256	19811967	0.08255	1.70E-05	0.144169	LPL
15	rs4777407	71826919	-0.06668	1.93E-05	0.351438	THSD4
2	rs11124147	107623734	-0.06382	1.97E-05	0.32488	intergenic

Chr: Chromosome; SNP: single nucleotide polymorphism. BP: Base position in the chromosome (Homo Sapiens GRCh37.p13 genome build used in Illumina HumanOmniExpress-24 BeadChip). Beta: regression coefficient that indicates the effect for the minor allele on DNAmTL; MAF: minor allele frequency. BMI: Body mass index in Kg/m^2.

[1]. p-value obtained in the multivariate regression model adjusted for sex, age, diabetes, and BMI for each SNP using a genetic additive approach.

antioxidants

MDPI

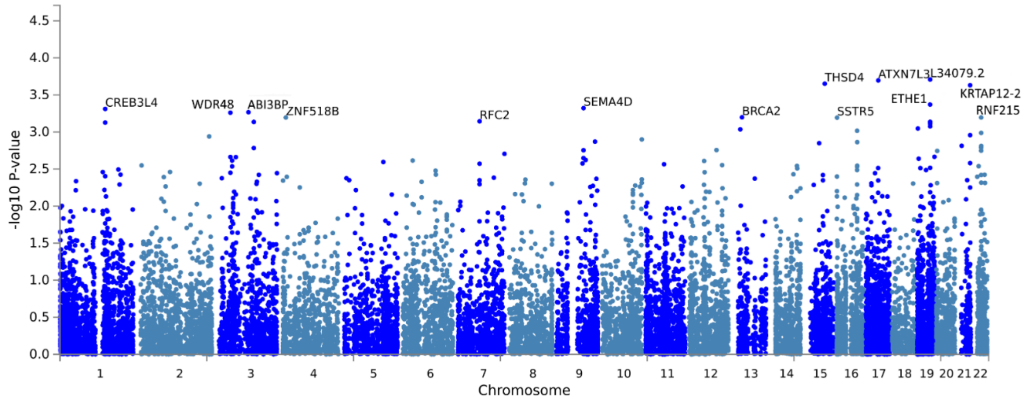

Figure S6. Manhattan plot for the gene-based GWAS analysis for telomere length-DNA methylation adjusted for age. The 14 top-ranked genes were annotated.

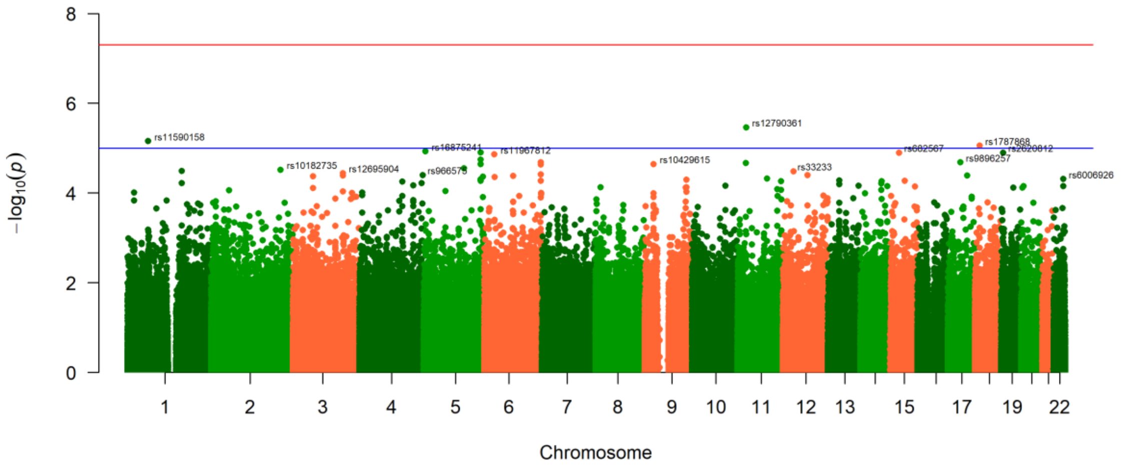

Figure S7. Manhattan plot of the SNP-based GWAS analizing the interaction between the SNPs and adherence to Mediterranean Diet (as dichotomous variable of 2 categories: low and high) in determining telomere length (DNAmTLAdjustAge). The top-ranked SNPs were annotated. The red line represents the threshold 1 ($-\log_{10}(5\times10^{-8})$) for the GWAS statistical significance. The blue line represents the threshold 2 ($-\log_{10}(1\times10^{-5})$).
